# TIMP‐1‐expressing breast tumor spheroids for the evaluation of drug penetration and efficacy

**DOI:** 10.1002/btm2.10286

**Published:** 2021-12-31

**Authors:** In Yeong Bae, Wooshik Choi, Seung Ja Oh, Chansoo Kim, Sang‐Heon Kim

**Affiliations:** ^1^ Center for Biomaterials, Biomedical Research Institute Korea Institute of Science and Technology (KIST) Seoul Republic of Korea; ^2^ Department of Biomedical Engineering, KIST school Korea University of Science and Technology Seoul Republic of Korea; ^3^ AI Laboratory, Computational Science Center and ESRI Korea Institute of Science and Technology Seoul Republic of Korea

**Keywords:** 3D multicellular tumor spheroid, adipose‐derived stromal cells, Collagen type‐1, extracellular matrix, multiple linear regression, tissue inhibitor of metalloproteinases‐1

## Abstract

Abundance of stromal cells and extracellular matrix (ECM) is observed in breast cancer, acting as a barrier for drug penetration and presenting a key issue for developing efficient therapeutics. In this study, we aimed to develop a three‐dimensional (3D) multicellular tumor model comprising cancer and stromal cells that could effectively mimic the drug resistance properties of breast cancer. Three different types of spheroid models were designed by co‐culturing breast cancer cells (MDA‐MB‐231) with three different types of stromal cells: human adipose‐derived stromal cells (hASCs), human bone marrow stromal cells, or human dermal fibroblasts. Compared with other models, in the hASC co‐culture model, tissue inhibitor of metalloproteinases‐1 (TIMP‐1) was highly expressed and the activity of matrix metalloproteinases was decreased, resulting in a higher ECM deposition on the spheroid surfaces. This spheroid model showed less drug penetration and treatment efficacy than the other models. TIMP‐1 silencing in hASCs reduced ECM protein expression and increased drug penetration and vulnerability. A quantitative structure–activity relationship study using multiple linear regression drew linear relationships between the chemical properties of drugs and experimentally determined permeability values. Drugs that did not match the drug‐likeness rules exhibited lower permeability in the 3D tumor model. Taken together, our findings indicate that this 3D multicellular tumor model may be used as a reliable platform for efficiently screening therapeutics agents for solid tumors.

## 1 INTRODUCTION

Breast cancer is one of the leading causes of cancer‐related deaths in women worldwide, and its incidence is increasing in all industrialized countries.^1^ It is a representative solid tumor, and the breast cancer tumor microenvironment (TME) consists of various cell types including tumor and stromal cells, surrounding extracellular matrix (ECM), and signaling molecules. The TME has recently emerged as a critical regulator of tumor progression and drug efficacy.^2^ It produces various cytokines and chemokines that regulate the composition and organization of ECM proteins. The ECM serves as a physical barrier, hindering drug entry into and diffusion throughout the tumor, thereby contributing to drug resistance.^3^, ^4^ In addition, interactions between cancer cells and stromal cells as well as various growth factors and ECM components in the TME can drastically affect the apoptotic sensitivity of cancer cells and their response to chemotherapeutic drugs.^5^, ^6^, ^7^

In recent years, interactions between stromal cells and cancer cells have been increasingly acknowledged to be involved in tumor development and progression. Emerging evidence indicates that stromal cells in the TME play important roles in ECM production.^8^ In breast cancer, tumor stromal cells such as cancer‐associated fibroblasts (CAFs) mainly contribute to abnormal ECM deposition in response to several paracrine factors such as reactive oxygen species (ROS), transforming growth factor‐beta (TGF‐β), and platelet‐derived growth factor (PDGF).^9^ In addition, the balance between matrix metalloproteinases (MMPs) and their endogenous inhibitors, tissue inhibitors of metalloproteinases (TIMPs), is a critical factor for stromal remodeling of the ECM.^10^ Many investigations have shown that among various isoforms of TIMPs, the expression of TIMP‐1 correlates with the progression and severity of several types of cancers, including colon cancer, lung carcinoma, gastric cancer, melanoma, and breast cancer.^11^, ^12^, ^13^, ^14^, ^15^ As a specific hallmark of human cancer, TIMP‐1 plays multiple roles related to the TME and drug resistance. Independent of its inhibitory effect on MMPs, TIMP‐1 acts as a growth factor that promotes cell proliferation and tumorigenesis in various cell types, including breast cancer cells.^11^, ^16^, ^17^ It may also function as a suppressor of apoptosis in several tumors.^18^, ^19^ Despite its diverse roles in breast cancer, it remains unclear whether TIMP‐1‐dependent ECM regulation affects the penetration or movement of drugs in cancer tissues.

Therefore, there is an urgent need to develop reliable platforms for screening effective therapeutic agents for breast cancer. Traditionally, the majority of cell‐based *in vitro* assays used to develop anticancer drugs in the pre‐clinical stage are facilitated using two‐dimensional (2D) cultures of cancer cells. However, this does not reflect the physiologic characteristics of three‐dimensional (3D) tumors in vivo. This has resulted in poor prediction of drug efficacy in vivo, leading to failures in clinical trials with huge financial losses.^20^ Thus, 3D culture models have recently attracted much attention as promising biotechnologies for more accurate drug efficacy screening. In contrast to 2D cell culture models, 3D tumor spheroid models recapitulate the physiologic features of human cancers in vivo in terms of heterogeneity, gene expression, signal pathways, cell–cell interactions, production/deposition of ECM, drug resistance and penetration, and 3D structure.^21^, ^22^, ^23^, ^24^ In addition, spheroid models are easy to control and are suitable for high‐throughput drug screening.^25^, ^26^

Recent technological advances have allowed for the development of several 3D tumor models that are utilized to understand cancer progression and inhibition.^27^, ^28^, ^29^ Specifically, 3D in vitro models that simulate the interaction between cancer cells and stromal cells have been designed for accurate evaluation of drug efficacy and penetration capacity.^30^, ^31^ However, few studies have focused on the diversity of stromal cell types used to generate 3D multicellular spheroids. To date, most research on 3D tumor models simulating the interaction between cancer cells and stromal cells has focused on using fibroblasts as a stromal component.^32^, ^33^, ^34^, ^35^ There remains a need for various types of 3D models that can be produced by incorporating other types of stromal cells to better understand anticancer drug resistance in the TME.

One of the important aspects of overcoming tumor drug resistance is understanding the physicochemical properties of therapeutic agents. Thus, researchers developed the notion of drug likeness, a rule that predicts whether a compound will possess the properties of a prototype drug in vivo based on its chemical properties during the early stages of drug development. Several considerations have been made to evaluate drug likeness. Analysis of the structures of drug candidates, pioneered by Lipinski with his Rule of Five (Ro5), has so far been a useful guide in achieving successful drug development.^36^ Modified measurements for drug‐likeness such as Veber's rule,^37^ Ghose filter,^38^ and quantitative estimate of drug‐likeness (QED)^39^ have been proposed; however, limitations still exist in predicting the actual bioavailability of drugs.^40^ Frequently used in vitro experiments such as parallel artificial membrane permeability assay (PAMPA), or Caco‐2 cell‐based assay, could not properly recapitulate drug resistance in solid tumors, since the properties of constituent membranes are significantly different.^41^, ^42^ To the best of our knowledge, there are few in vitro models used for evaluating drug permeability and resistance in solid tumors.^43^

In this study, we aimed to develop 3D multicellular tumor spheroid models comprising cancer cells and human adipose‐derived stromal cells (hASCs) that could accurately evaluate drug efficacy and resistance. We also investigated and compared the morphological characteristics, as well as physical and biochemical properties of the models in relation to drug responses.

## 2 RESULTS

### 2.1 Morphological characteristics of tumor spheroids with different types of stromal cells

**FIGURE 1:**
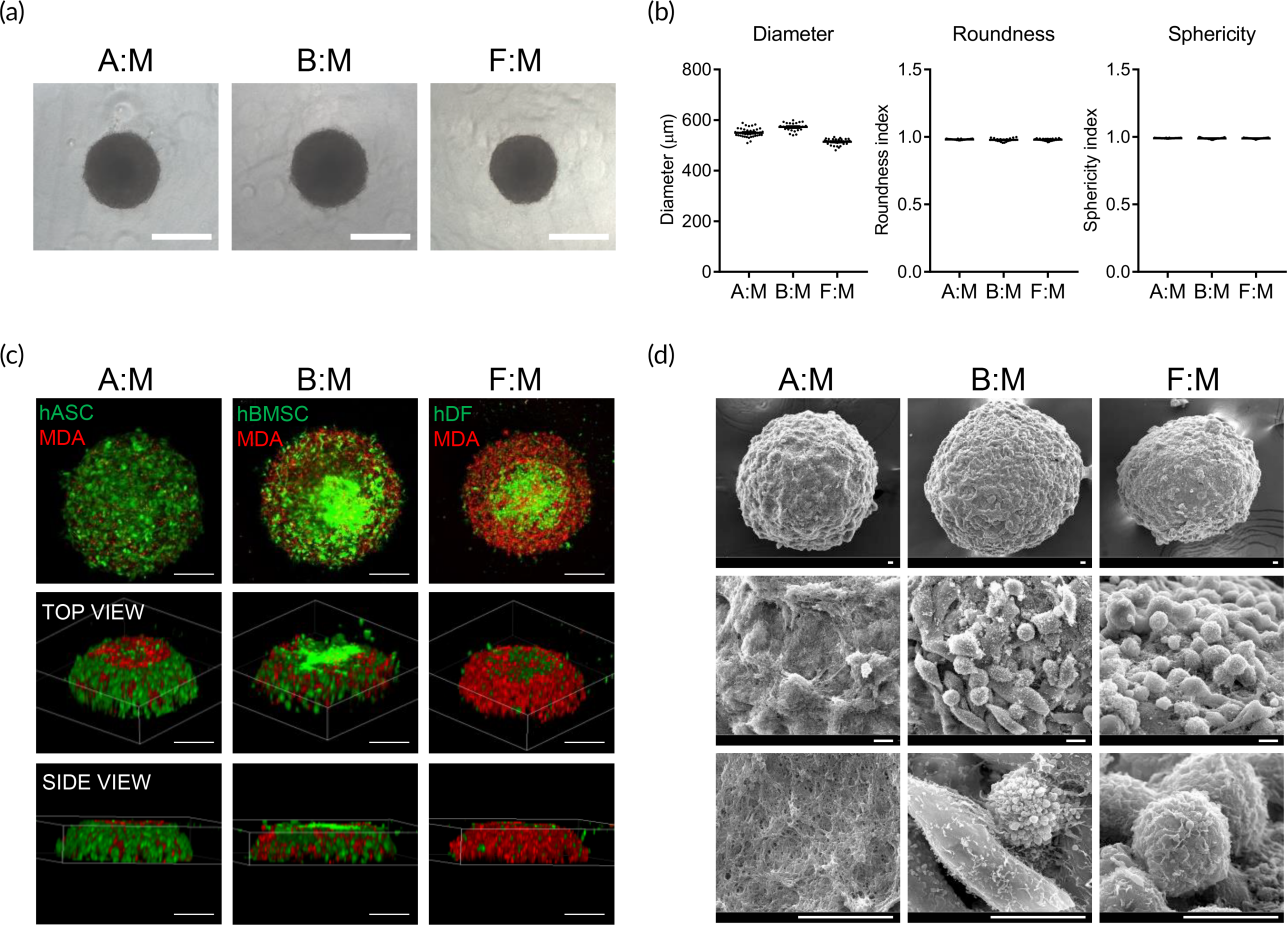
Formation and morphological characterization of 3D multicellular tumor spheroids. (a) Representative phase‐contrast images of tumor spheroids at 2 days after seeding with densities of 10,000 cells/well. Scale bar, 500 μm. (b) Shape properties of tumor spheroids. *n* = 40 per group. (c) Distribution of cancer cells and stromal cells in the tumor spheroids. Stromal cells (hASCs, hBMSCs, or hDFs) and breast cancer cells (MDA‐MB‐231) were stained with green and red fluorescent dyes, respectively. Scale bars, 200 μm. (d) SEM images of tumor spheroids, scale bars, 10 μm. A:M, B:M, or F:M; multicellular tumor spheroids generated using hASC, hBMSC, or hDF along with MDA‐MB‐231, respectively. 3D, three dimension; hASCs, human adipose‐derived stromal cells; hBMSCs, human bone marrow stromal cells; hDFs, human dermal fibroblasts; SEM, standard error of the mean

To mimic the interaction between stromal cells and cancer cells in an in vivo TME, we engineered 3D multicellular tumor spheroids by co‐culturing breast cancer cells and stromal cells (Figure 1a). MDA‐MB‐231, a breast carcinoma cell line, was used to represent the breast cancer cells. Among the various stromal cell types, hASCs, human bone marrow stromal cells (hBMSCs), and human dermal fibroblasts (hDFs) were selected because they could comprise the stromal cell population of breast tumors.^8^ To form 3D multicellular tumor spheroids, each stromal cell type was cultured with MDA‐MB‐231 cells in a 1:1 ratio on poly(2‐hydroxyethyl methacrylate) (poly‐HEMA)‐coated plates with 5% Matrigel. After 48 h of culture, 3D multicellular tumor spheroids were formed through the self‐organization of single cells in the plates (Figure 1a). Multicellular tumor spheroids generated using hASCs, hBMSCs, or hDFs along with MDA‐MB‐231 are indicated as A:M, B:M, or F:M in the figures, respectively.

To investigate the morphologic characteristics of 3D multicellular tumor spheroids, several shape parameters, such as diameter, roundness, and sphericity, were analyzed from phase‐contrast images. As shown in Figure 1b, all spheroids with different stromal cells were uniformly rounded and spherical, with diameters ranging from 500 to 600 μm. These data indicate that all spheroids were consistently and uniformly generated as 3D well‐defined geometrical shapes.

Next, we investigated the local distribution of breast cancer cells and stromal cells in each multicellular spheroid. Stromal and breast cancer cells were stained with green and red fluorescent dyes, respectively, and were cultured to form multicellular spheroids. In hASC‐co‐cultured tumor spheroids, breast cancer cells were mainly positioned in the interior of the spheroids, while most of the hASCs were distributed on the surface of the spheroids. In contrast, in hDF‐co‐cultured tumor spheroids, the majority of breast cancer cells were located on the surface of the spheroids. In hBMSC‐co‐cultured spheroids, cancer and stromal cells were evenly distributed (Figure 1c). The surface structures of multicellular spheroids were analyzed using scanning electron microscopy (SEM). In hBMSC‐ and hDF‐co‐cultured tumor spheroids, the surfaces were relatively rough and mostly composed of cells. On the other hand, the surface of hASC‐co‐cultured spheroids mainly consisted of ECM components, and cells were hardly found on the surface (Figure 1d). Considering that the distribution of stromal cells and ECM on the surface of tumor tissue is one of the major characteristics found in in vivo breast cancer,^44^ the results suggest that hASC‐co‐cultured spheroids mimic the in vivo tumor environment better than the other models.

### 2.2 hASC‐co‐cultured tumor spheroids showed higher ECM protein expression than other models

**FIGURE 2:**
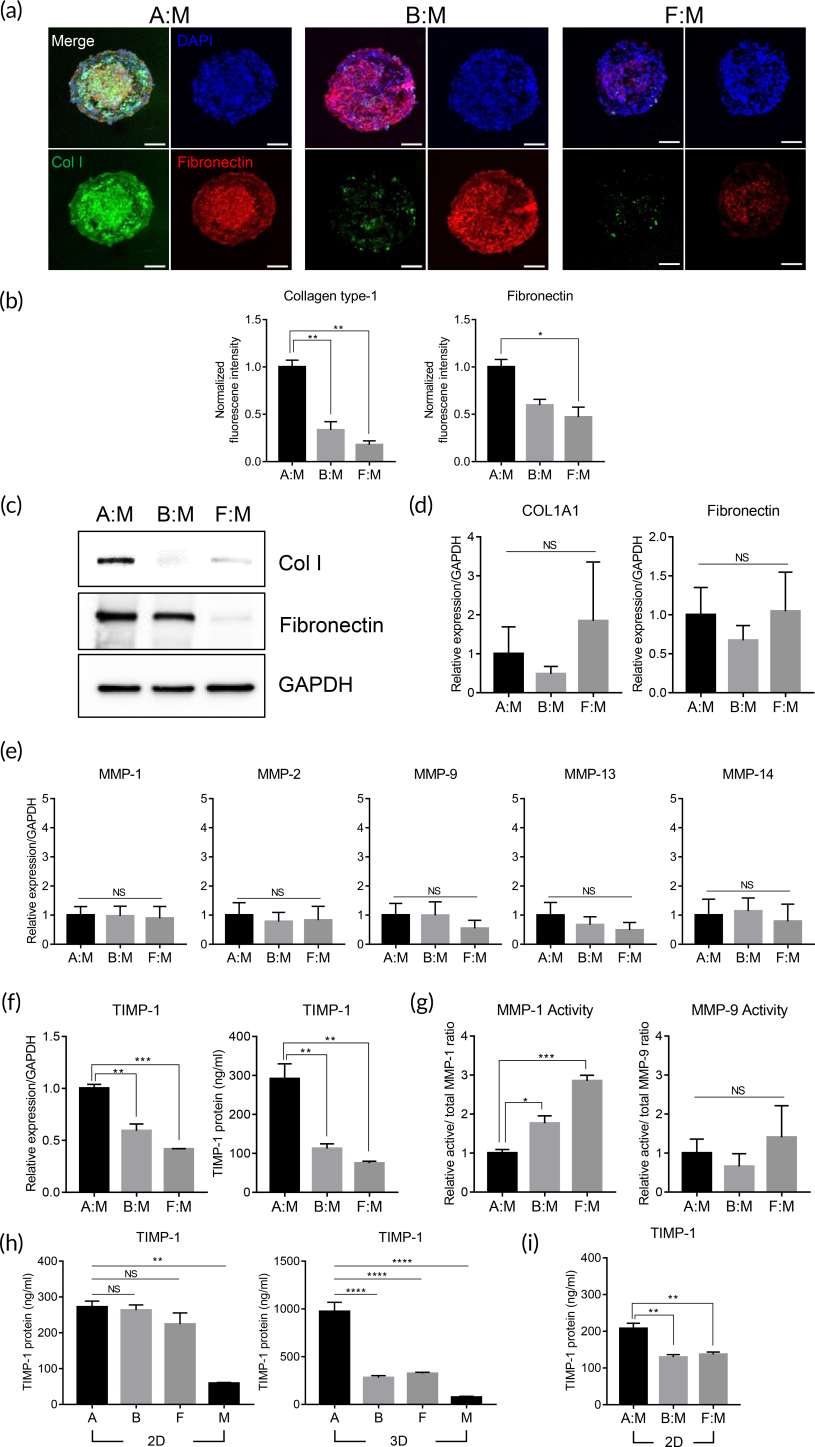
Effect of TIMP‐1 and MMPs on ECM expression in tumor spheroids. (a) Collagen type‐1 (green) and fibronectin (red) staining of tumor spheroids A:M, B:M, and F:M. Scale bars, 100 μm. (b) Quantifications of fluorescence intensity for Collagen type‐1 and fibronectin in the tumor spheroids. Values were normalized to the intensity of DAPI. *p < 0.05, **p < 0.01 (one‐way ANOVA), *n* = 3 per group. (c) Protein expression of collagen type‐1 and fibronectin in the tumor spheroids. mRNA expression of (d) Collagen type‐1 alpha 1, fibronectin, and (e) MMPs in the tumor spheroids (one‐way ANOVA), *n* = 3 per group. (f) mRNA expression and protein secretion of TIMP‐1 in the tumor spheroids. **p < 0.01, ***p < 0.001 (one‐way ANOVA), *n* = 3 per group. (g) Enzymatic activity of MMP‐1 and MMP‐9 secreted from the tumor spheroids. *p < 0.05, ***p < 0.001 (one‐way ANOVA), *n* = 3 per group. (h) TIMP‐1 protein secreted from 2D‐ and 3D‐cultured stromal cells and breast cancer cells. **p < 0.01, ****p < 0.0001 (one‐way ANOVA), *n* = 3 per group. (i) TIMP‐1 protein secreted from 2D‐co‐cultured stromal cells and breast cancer cells. **p < 0.01 (one‐way ANOVA), *n* = 3 per group. For RT‐qPCR analysis, values were normalized to GAPDH. All data are presented as mean ± SEM. Col I, Col1a1, 3D—a, b, f, or m; monocellular spheroid of hASCs, hBMSCs, hDFs, or MDA‐MB‐231, respectively. 2D—a, b, f, or m; monolayer culture of hASCs, hBMSCs, hDFs, or MDA‐MB‐231, respectively. 2D, two dimension; 3D, three dimension; DAPI, 4′,6‐diamidino‐2‐phenylindole; Col I, Collagen type‐1; Col1a1, Collagen type‐1 alpha 1; ECM, extracellular matrix; hASCs, human adipose‐derived stromal cells; hBMSCs, human bone marrow stromal cells; hDFs, human dermal fibroblasts; SEM, standard error of the mean; MMPs, metalloproteinases; NS, not significant; TIMP‐1, tissue inhibitor of metalloproteinases‐1

To confirm whether ECM protein expression was higher in hASC‐co‐cultured tumor spheroids than in other models, 3D multicellular spheroids were analyzed via immunofluorescence using antibodies against Collagen type I (Col I) and fibronectin, which are two major ECM components found in breast cancer.^45^ Spheroids with hASCs and MDA‐MB‐231 showed significantly higher expression of Col I and fibronectin than the hBMSC‐ or hDF‐co‐culture model (Figure 2a,b). When spheroids were formed with a single type of cell, Col I and fibronectin were rarely expressed (Figure S1a–c). Western blot analysis also confirmed that Col I and fibronectin expression was higher in the hASC co‐culture model than in the other models (Figure 2c). These results indicate that ECM genes were highly expressed in spheroids wherein hASCs and breast cancer cells could interact.

### 2.3 ECM overexpression in the hASC‐co‐cultured spheroid model is associated with the TIMP‐1 upregulation

ECM protein expression is regulated by a balance between protein synthesis and degradation. To understand the mechanism(s) underlying the high ECM protein expression in the hASC‐co‐cultured tumor spheroid model, we analyzed the factors related to ECM synthesis and degradation. RNA and conditioned media from spheroids were prepared and subjected to quantitative reverse transcription polymerase chain reaction (RT‐qPCR) and enzyme‐linked immunosorbent assay (ELISA), respectively. The RNA levels of Col I and fibronectin, the major ECM components of the spheroids, were not significantly different among the spheroid models (Figure 2d). In addition, TGF‐β, which is known to play a major role in ECM synthesis, showed similar RNA and protein expression levels among all spheroids (Figure S2a,b). These data imply that ECM protein expression might be controlled at the posttranscriptional level.

ECM protein degradation is mainly regulated by the interplay between MMPs and their endogenous inhibitor, TIMPs. Among various MMPs, collagenases (MMP‐1, MMP‐13, and MMP‐14) and gelatinases (MMP‐2 and MMP‐9) mainly contribute to the degradation of ECM proteins.^46^ Thus, the expression levels of collagenases and gelatinases were investigated using RT‐qPCR. The RNA levels of these genes were not significantly different among spheroid models (Figures 2e and S1d). On the other hand, TIMP‐1, which is mainly expressed in breast cancer, was highly upregulated in hASC‐co‐cultured spheroids at both the RNA and protein levels compared to the other models (Figure 2f). Since MMP‐1 and MMP‐9 have been reported to play crucial roles in breast cancer,^47^ the activities of MMP‐1 and MMP‐9 in spheroid‐conditioned media were also analyzed. Following the expression profile of TIMP‐1, the activity of MMP‐1 was significantly lower in spheroids with hASCs and breast cancer cells than in spheroids from other models, while the activity of MMP‐9 was similar among spheroids (Figure 2g).

To investigate the factors that affect the increased expression of TIMP‐1 in the hASC co‐culture model, we analyzed the TIMP‐1 expression in stromal cells under various conditions, including culturing as a monolayer, 3D spheroid culture, and co‐culturing with breast cancer cells. In monolayer culture, stromal cells showed a higher TIMP‐1 protein expression than breast cancer cells. There were no significant differences among stromal cells (Figure 2h). When hASCs were cultured in the 3D spherical configuration, TIMP‐1 expression was increased by approximately fourfold compared to that in the monolayer culture; however, no significant changes were observed in other stromal cells (Figure 2h). The effect of co‐culturing with stromal and breast cancer cells on TIMP‐1 expression was also investigated. When hASCs were co‐cultured with breast cancer cells, TIMP‐1 protein expression was increased by approximately 1.25 times compared to that when the two cells were cultured separately. In the case of hBMSCs or hDFs, co‐culturing with breast cancer cells did not have a significant effect on TIMP‐1 protein levels (Figure 2h,i). The RNA level of TIMP‐1 showed a similar pattern; *TIMP‐1* expression in hASCs was significantly increased by the 3D spheroid formation and co‐culturing with breast cancer cells, as compared to that in hBMSCs and hDFs (Figure S3a–c). Taken together, these results suggest that TIMP‐1 overexpression in hASC co‐cultured spheroids is involved in the regulation of MMP‐1 activity and ECM expression.

### 2.4 Tumor spheroids co‐cultured with hASC demonstrated lower drug penetration and efficacy

**FIGURE 3:**
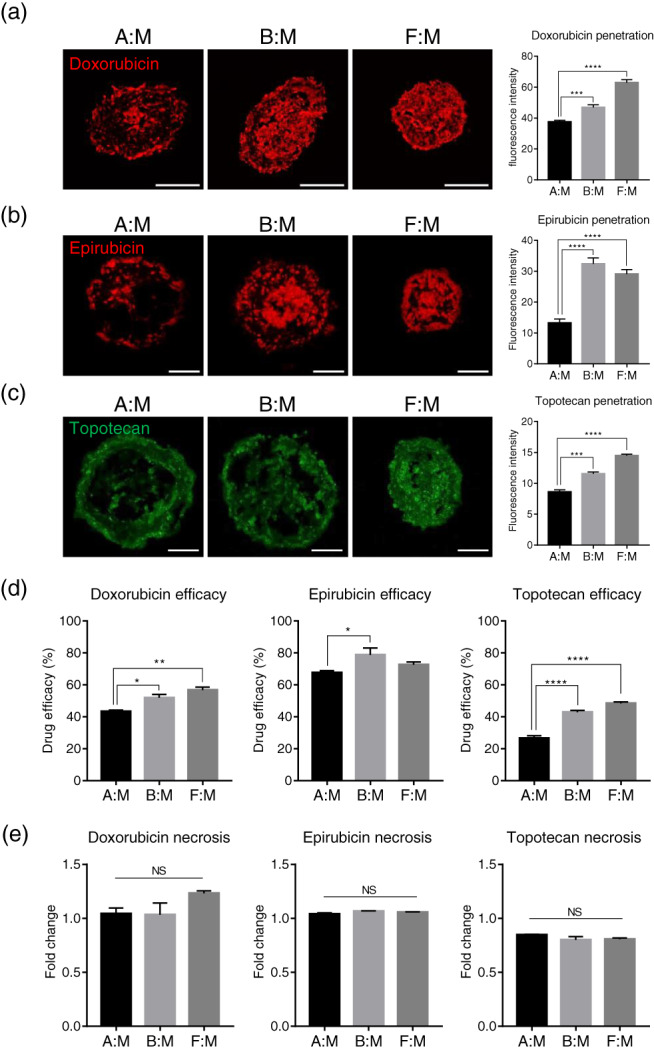
Effects of anticancer drugs on the survival of 3D multicellular tumor spheroids. Stromal cells and the breast cancer cells were co‐cultured to form tumor spheroids for 48 h, and anticancer drugs were treated to the spheroids for an additional 48 h. Disposition of (a) doxorubicin, (b) epirubicin, and (c) topotecan penetrated into the tumor spheroids. Quantifications of fluorescence intensity for anticancer drugs were indicated in the graph. ***p < 0.001, ****p < 0.0001 (one‐way ANOVA), *n* = 3 per group. Scale bars, 200 μm (a) and 100 μm (b) and (c). (d) Efficacy of anticancer drugs in the tumor spheroids. *p < 0.05, **p < 0.01, ****p < 0.0001 (one‐way ANOVA), *n* = 3 per group. (e) Relative fold changes of necrotic cells in tumor spheroids by anticancer drug treatment. Necrotic cells in the tumor spheroids before drug treatment are presented as 1. *n* = 3 per group. All data are presented as mean ± SEM. 3D, three dimension; ANOVA, analysis of variance; NS, not significant; SEM, standard error of the mean; TIMP‐1, tissue inhibitor of metalloproteinases‐1

We next tested whether ECM deposition on the surface of spheroids would affect the penetration of anticancer drugs and their efficacy. Three types of 3D multicellular tumor spheroids were formed and treated with 10 μM doxorubicin, epirubicin, or topotecan, which are chemotherapeutic agents for various tumors, including breast cancer, for an additional 48 h. Since these chemical compounds were reported to have intrinsic fluorescence, the distribution of drugs inside the spheroids was analyzed via fluorescence microscopy. Doxorubicin, epirubicin, and topotecan were distributed throughout the treated hBMSC‐ or hDF‐co‐cultured spheroids. In contrast, there was a significant reduction in the amounts of these anticancer drugs inside the hASC‐co‐cultured spheroids, as compared to those in other spheroids (Figure 3a–c). When spheroids were formed with a single‐cell type, the drugs were found to penetrate all types of spheroids (Figure S4a,b). These results suggest that drug penetration is lowered specifically in the hASC‐co‐cultured tumor model.

Next, we investigated whether drug penetration is correlated with drug response in 3D multicellular tumor spheroids. The viability of spheroids and the presence of necrosis following anticancer drug treatment were measured and analyzed. Drug efficacy (ratio of nonviable cells to drug treatment as determined by Equation (3)) was significantly decreased in hASC‐co‐cultured spheroids compared to the other models (Figure 3d). Very few necrotic cells were observed in all types of 3D multicellular tumor spheroids (Figure 3e). These data indicate that the lowered drug penetration in hASC‐co‐cultured spheroids may contribute to the drug resistance properties of this tumor model.

### 2.5 TIMP‐1 silencing in hASC‐co‐cultured tumor spheroids affects ECM protein expression and drug efficacy

**FIGURE 4:**
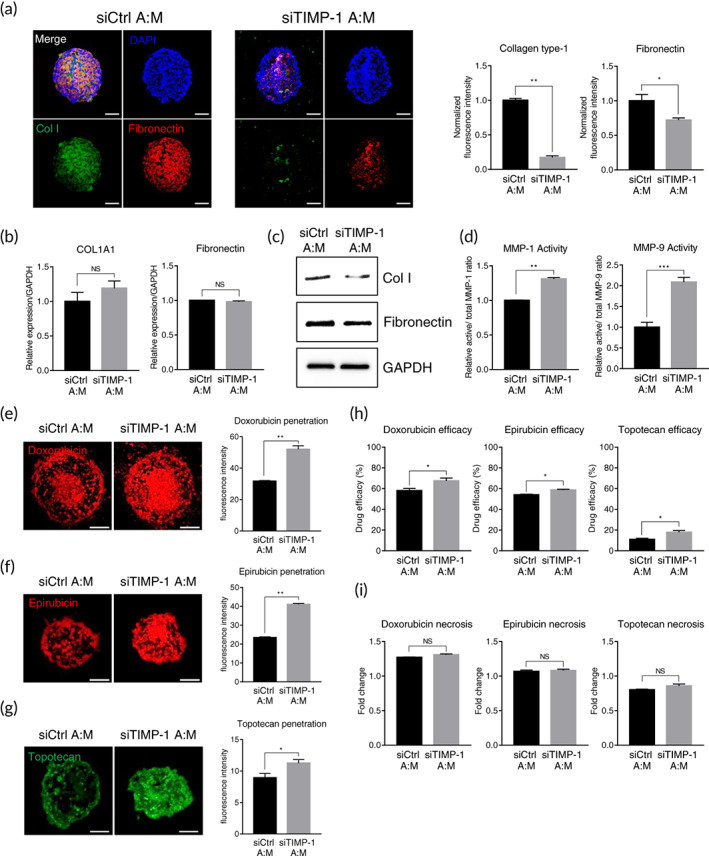
Effects of TIMP‐1 silencing in hASC on ECM expression and drug efficacy in tumor spheroids. hASCs were transfected with TIMP‐1 or control siRNA and co‐cultured with breast cancer cells for 48 h to form tumor spheroids. (a) Collagen type‐1 (green) and fibronectin (red) staining of tumor spheroids. Scale bars, 100 μm. Quantifications of fluorescence intensity for collagen type‐1 and fibronectin were indicated in the graph. Values were normalized to the intensity of DAPI. *p < 0.05, **p < 0.01, *n* = 3 per group. Effect of TIMP‐1 silencing on (b) the mRNA and (c) the protein expression of ECM markers in the tumor spheroids (unpaired student's *t*‐test), *n* = 3 per group. (d) Effect of TIMP‐1 silencing on the activity of MMP‐1 and MMP‐9 in the tumor spheroids. **p < 0.01, ***p < 0.001, *n* = 3 per group. (e–i) The tumor spheroids generated with TIMP‐1 silenced hASCs and breast cancer cells were treated with anticancer drugs for an additional 48 h. Effect of TIMP‐1 silencing on the disposition of (e) doxorubicin, (f) epirubicin, and (g) topotecan in the tumor spheroids. Quantifications of fluorescence intensity for anticancer drugs were indicated in the graph. *p < 0.05, **p < 0.01, *n* = 3 per group. Scale bars, 200 μm. (h) Effect of TIMP‐1 silencing on the efficacy of anticancer drugs in the tumor spheroids. *p < 0.05, *n* = 3 per group. (i) Effect on the necrosis of anticancer drugs in the tumor spheroids. Necrotic cells in the tumor spheroids before drug treatment are presented as 1. NS, not significant, *n* = 3 per group. All data are presented as mean ± SEM, and the statistical significances were determined by unpaired student's *t*‐test. DAPI, 4′,6‐diamidino‐2‐phenylindole, ECM, extracellular matrix; hASCs, human adipose‐derived stromal cells; MMPs, metalloproteinases; NS, not significant; TIMP‐1, tissue inhibitor of metalloproteinases‐1; SEM, standard error of the mean

To test whether TIMP‐1 mainly affects the increased ECM protein expression in hASC‐co‐cultured spheroids, hASCs were transfected with either control siRNA or siRNA against *TIMP‐1*, followed by the formation of spheroids with MDA‐MB‐231 cells. The distribution of cancer cells and stromal cells in tumor spheroids did not change significantly with TIMP‐1 silencing (Figure S2c). Spheroids were prepared, and components of the ECM were analyzed using RT‐qPCR, western blotting, and immunofluorescence. The TIMP‐1 RNA level was greatly reduced by siRNA transfection in both monolayer cells and 3D spheroids (Figure S2d,e). TIMP‐1 silencing in hASC‐co‐cultured spheroids did not significantly affect the RNA levels of ECM components, such as Col I and fibronectin (Figure 4b). However, when hASC‐co‐cultured spheroids were transfected with siTIMP‐1, the protein levels of Col I and fibronectin were drastically reduced compared to the control spheroids (Figure 4a,c). The effect of TIMP‐1 knockdown in hASCs on MMP activity was also analyzed. In tumor spheroids co‐cultured with TIMP‐1‐silenced hASCs, the activities of MMP‐1 and MMP‐9 were significantly higher than those in the control (Figure 4d). Overall, our data show that TIMP‐1 in hASCs plays a major role in the expression of ECM proteins in tumor spheroids by regulating MMP‐1 activity.

**FIGURE 5:**
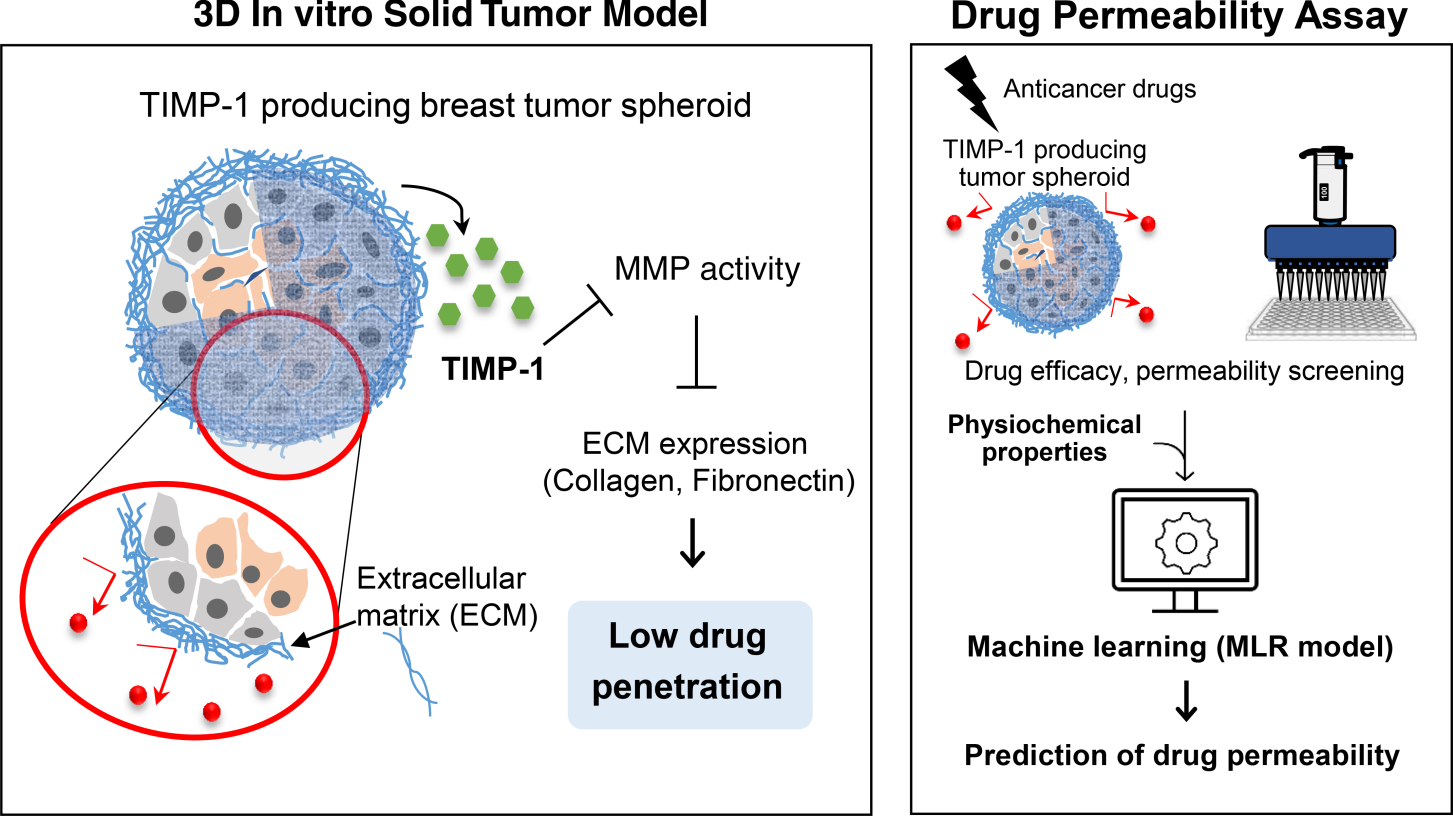
Schematic illustration of TIMP‐1 mechanism of action in hASC‐co‐cultured tumor model. Proposed model for TIMP‐1 function on ECM expression and drug resistance in hASC‐co‐cultured tumor spheroids. TIMP‐1 was upregulated during multicellular spheroid formation and the activity of MMPs was reduced, resulting in high ECM deposition on the surface of the spheroids. TIMP‐1 expressed in this 3D tumor model reduced drug penetration and treatment efficacy. 3D, three dimension; ECM, extracellular matrix; hASCs, human adipose‐derived stromal cells; MMPs, metalloproteinases; TIMP‐1, tissue inhibitor of metalloproteinases‐1

The effects of TIMP‐1 silencing on the drug response of spheroids were also investigated. hASCs were transfected with control siRNA or siTIMP‐1 and co‐cultured with MDA‐MB‐231 to form tumor spheroids followed by three anticancer drug treatments: doxorubicin, epirubicin, and topotecan. The distribution of each chemotherapeutic agent inside spheroids was further analyzed. In the hASC‐co‐cultured model, TIMP‐1 silencing significantly increased the amount of drug penetrating the spheroids, as compared with the control (Figure 4e–g). The effects of TIMP‐1 on drug efficacy and necrosis were also evaluated. Tumor spheroids co‐cultured with TIMP‐1‐silenced hASCs showed significantly increased drug efficacy compared to that of the control (Figure 4h). Few necrotic cells were observed in both spheroids for all three drugs (Figure 4i). Taken together, these results indicate that TIMP‐1 in hASCs could be involved in the process of drug penetration and resistance in our 3D tumor model (Figure 5).

### 2.6 Drug permeability in tumor spheroids correlates with Caco‐2, PAMPAs, and drug‐likeness rules

**FIGURE 6:**
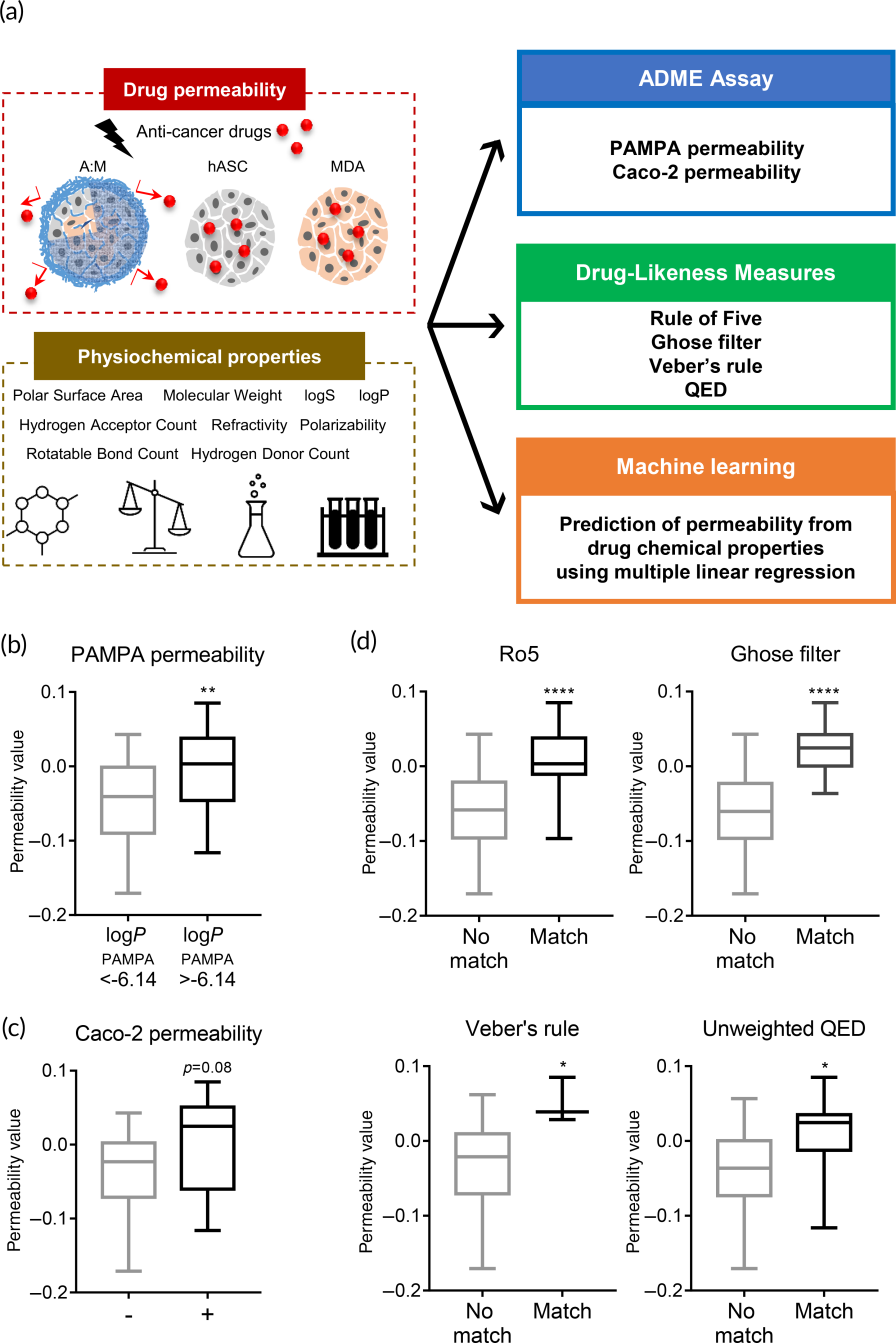
Correlation analysis of permeability values in the tumor spheroids. (a) Schematic overview of drug screening analyses using 16 anticancer drugs. PVs were calculated with the efficacy of anticancer drugs in A:M tumor spheroids, monocellular spheroids with either hASCs or breast cancer cells as described in Equation (4). (b) Comparison of PVs of the tumor spheroids depending on (b) *Log*P_PAMPA_ and (c) CaCo‐2 monolayer penetrability. **p < 0.01 (unpaired student's *t*‐test). Effect of (d) Ro5, (e) Ghose filter, (f) Veber's rule, and (g) unweighted QED compliance of drugs on the PVs of the tumor spheroids. *p < 0.05, ****p < 0.0001 (unpaired student's *t*‐test). All data are presented as mean ± SEM. PVs of 16 drugs are listed in Table S2. hASCs, human adipose‐derived stromal cells; ADME, absorption, distribution, metabolism and elimination; PVs, permeability values; QED, quantitative estimate of drug‐likeness; SEM, standard error of the mean

We further tested whether the 3D tumor model inhibited the penetration of drugs other than doxorubicin, epirubicin, and topotecan. Table S1 presents 16 anticancer drugs and their half‐maximal inhibitory concentration (IC_50_) values in MDA‐MB‐231 cells. These compounds reportedly act inside the nucleus and have similar mechanisms to prevent the division and proliferation of cancer cells. As shown in Figure 6a, the drug efficacy of 16 compounds was tested in three types of spheroids: hASC and MDA‐MB‐231 co‐cultured spheroids, and monocellular spheroids formed with either hASCs or breast cancer cells, respectively. If the drug efficacy in multicellular spheroids was lower than the average efficacy in hASC and MDA‐MB‐231 monocellular spheroids, it might be recognized that drug penetration in multicellular spheroids was disturbed. Therefore, we defined the permeability value (PV) of each drug as the difference in efficacy in multicellular and monocellular spheroids, as determined by Equation (4). We further investigated whether PV would correlate with canonical indicators of drug permeability, such as the PAMPA, Caco‐2 permeability assay, and drug‐likeness rules (Figure 6a). As shown in Figure 6b, drugs with log*P*_PAMPA_ less than −6.14, which is reported to have a low permeability,^48^ had significantly lower PVs than drugs with log*P*_PAMPA_ values over −6.14. In addition, drugs that were impermeable to Caco‐2 monolayer cells tended to show lower PVs in the tumor model compared to the permeable drugs (Figure 6c).

Next, we analyzed the correlations between the drug‐likeness rules and PVs. Drug‐likeness rules are simple predictions of how the physicochemical properties of a compound would affect molecular kinetics; in particular, permeability, solubility, and metabolic stability in vivo. For widely used rules such as Ro5, Ghose filter, Veber's rule, and unweighted QED, drugs satisfying each of the drug‐likeness rules had significantly higher PVs in the tumor model than those that did not match (Figure 6d). These results indicate that the PVs derived from our 3D tumor model had strong correlations with the values for evaluating drug permeability.

### 2.7 Quantitative structure–activity relationship analysis for predicting drug permeability using multiple linear regression

**FIGURE 7:**
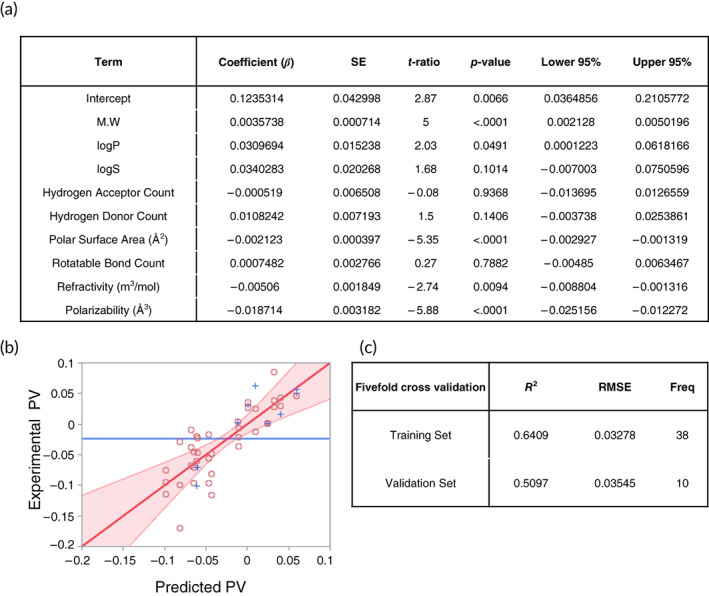
QSAR analysis for predicting drug permeability. (a) Chemical properties and its coefficients affecting PVs in the tumor spheroids derived from MLR. (b) Scatter plot obtained from the comparison of experimental PVs and predicted PVs in the training set. Circles and crosses indicate training and validation data set, respectively. Red area represented the values with *p* < 0.05. (c) Coefficient of determination and RMSE values in the training and validation set from cross‐validation. Variants of 16 drugs derived from MLR are presented in Table S3. MLR, multiple linear regression; PVs, permeability values; QSAR, quantitative structure–activity relationship

To investigate the relationship between PVs and the chemical properties of drugs and thus predict PVs based on their chemical properties, we performed multiple linear regression (MLR) analysis. Multiple regression is a widely accepted statistical technique that utilizes several explanatory variables (independent variables) to predict the outcome of a response variable. Table S4 shows the values of the chemical properties of the drugs (independent variables) and their corresponding experimental PVs (output variable). We assumed that there were no instrumental variables or nonlinear terms, because we observed mere nominal interactions among the chemical properties. We divided the overall dataset (total 48 sets) into 2 categories with proportions of 80% and 20%, which are of the Pareto rule: the training set (39 sets) and the validation set (9 sets). We also applied fivefold cross‐validation for robustness. Normally, more than 30 data sets are considered sufficient for Gaussian analysis, and linear regression is performed well under the Gaussian assumption. Forward stepwise MLR analysis revealed nine descriptors used to establish the training set, and the following equation was obtained:

(1) $$\hat{\mathrm{PV}}=\alpha +\beta K+\gamma L+\delta M+\varepsilon N+\mathrm{\zeta O}+\mathrm{\eta P}+\mathrm{\theta Q}+\mathrm{\kappa R}+\mathrm{\lambda S},$$

where $\hat{\mathrm{PV}}$ is the predicted PV, *K* is the molecular weight (MW), L is log*P*, *M* is log*S*, *N* is the hydrogen acceptor count, *O* is the hydrogen donor count, *P* is the polar surface area (PSA), *Q* is the rotatable bond count, *R* is the refractivity, *S* is the polarizability, and α to λ are the regression coefficients. Logarithmic values were applied to represent the rate of change. The regression coefficients are as follows: α=0.1235, β=0.0036, γ=0.0309, δ=0.0340, ε=−0.0005, ζ=0.0108, η=−0.0021, θ=0.0007, κ=0.0051, and λ=−0.0187 (Figure 7a).As shown in Figure 7a, five out of nine independent variables, which are the descriptors, *K*, log*P*, *P*, *R*, and *S*, are found to significantly affect the prediction of PV in MLR analysis. Specifically, molecular weight (*K*) and log*P* have positive regression coefficients, showing that PV is proportional to them, while PSA (*P*), refractivity (*R*), and polarizability (*S*) showed negative regression coefficients (Figure 7a). It was observed that the MLR analysis was statistically significant (*p* < 0.001). A positive correlation was also found between the predicted PV ($\hat{\mathrm{PV}}$) and experimental PV (*R* ^2^ = 0.69, root mean square error (RMSE) = 0.0347) (Figure 7b). We validated our quantitative structure–activity relationship (QSAR) MLR model with fivefold cross‐validation by randomly splitting data objects into five disjoint equivalent subsets. Model regression was performed with four subsets (38 or 39 training data sets) and was validated using the remaining 1 subset. This process was repeated five times for cross‐validation. The fact that the validation data set showed a comparable level of determination constants (*R* ^2^ = 0.51 and RMSE = 0.0355) to the training data set (*R* ^2^ = 0.64 and RMSE = 0.0328) confirms the versatility of the QSAR MLR model in predicting drug permeability (Figure 7c). Taken together, these results suggest that 3D tumor models co‐culturing breast cancer cells and hASCs could effectively mimic the drug‐resistant features of in vivo tumors and that the QSAR approach to this model might be an efficient tool for screening permeable compounds in the early stages of drug development.

## 3 DISCUSSION

In this study, we demonstrated that spheroids co‐cultured with breast cancer cells and hASCs are suitable as in vitro 3D multicellular tumor models for drug screening. Among the three different types of spheroids (MDA‐MB‐231 co‐cultured with hASCs, hBMSCs, or hDFs), breast cancer cells co‐cultured with hASCs had a higher content of ECM components on the surface of spheroids and showed lower drug efficacy than when co‐cultured with the other stromal cells. Data from the spheroids with TIMP‐1‐silenced hASC and breast cancer cells indicated that TIMP‐1 might play a key role in the regulation of ECM protein expression. A machine learning approach for drug screening based on multiple regression analysis revealed that this tumor spheroid model could recapitulate the penetration efficiency of drugs determined by chemical features and various drug‐likeness methods. Taken together, breast cancer and hASC clusters in the form of 3D spheroids may be a reliable model for screening efficient therapeutics used to treat solid tumors.

In breast cancer in vivo, the TME consists of ECM and stromal cells, such as fibroblasts, mesenchymal stromal cells (MSCs), immune cells, and vascular endothelial cells.^49^ Stromal tissue in the TME reportedly plays an important role in cancer development and progression. Stromal tissues in TME have been reported to play an important role in cancer development and progression. Therefore, tumor–stromal ratio (TSR), a proportion of the stromal region inside the TME, has been identified as a promising parameter for cancer prognostication.^50^ Clinically, patients with more than 50% TSR are classified as stroma rich, whereas those with less than 50% TSR classified as stroma poor: the two groups are significantly different in survival and prognosis.^51^ To evaluate both populations without bias, we chose a ratio of 1:1 for cancer and stromal cells to generate a 3D tumor model. In this study, we focused on the interaction of hASCs and breast cancer cells and their ability to produce ECM, which thus provides drug resistance. We found that TIMP‐1 expressed in hASCs regulated the deposition of ECM on the surface of spheroids, as well as the penetration of drugs into the 3D tumor model. In addition, several studies have investigated the effects of hASCs on the TME of breast cancer. Studies have shown that the secretion of various factors, including leptin, insulin‐like growth factor 1, and oncostatin M, from hASCs could promote breast cancer.^52^ Recently, breast cancer cells have been shown to stimulate hASCs to secrete cytokines by upregulating the expression of C‐X‐C ligand 5 (CXCL5)^53^ or S100 calcium‐binding protein A7 (S100A7), a small calcium‐binding protein.^54^ These findings suggest that hASCs interact with breast cancer cells and are involved in the development of breast cancer through a variety of mechanisms. Moreover, our 3D tumor model may be able to mimic several characteristics of in vivo breast cancer, such as 3D morphology, hypoxic environment in its core region, stromal cell distribution, surface ECM deposition, and drug resistance; thus, our model could potentially provide more reliable drug screening results compared to existing ones.

The amount of ECM that accumulates locally is determined by the balance between the synthesis and degradation of ECM components. We found that our 3D tumor model involving the co‐culturing of hASCs and breast cancer cells showed increased ECM protein deposition on the surface due to TIMP‐1 overexpression, thereby inhibiting ECM degradation. However, the ECM protein expression in hASC monocellular spheroids was much lower, even though the TIMP‐1 level was comparable to that in multicellular models. (Figures S4a and 2h). These data imply that the agents derived from breast cancer are responsible for ECM synthesis. Song et al. also demonstrated that the agents in the extracellular vesicles of breast cancer cells convert hASCs into myofibroblasts, thereby promoting ECM remodeling.^55^ Factors such as ROS, lysophosphatidic acid, TGF‐β, and PDGF are all known to be involved in the recruitment and activation of CAFs.^56^ Among these, TGF‐β plays a key role in the synthesis of ECM; it binds to its membrane receptor and activates downstream signaling molecules such as Smad2 and Smad3. Activated Smad protein complexes can translocate to the nucleus and act as transcription factors for various ECM genes.^57^ However, TGF‐β seemed to play a minor role in our case, since the RNA and protein levels of TGF‐β were not significantly different among various stromal cells and breast cancer cells (Figure S2a,b). The factors that contribute to ECM synthesis in breast cancer need to be elucidated. It might be possible to inhibit ECM deposition in solid tumors more efficiently by co‐regulating the ECM‐promoting factor and TIMP‐1, which inhibits ECM degradation. This may allow for increased penetration and efficacy of anticancer drugs inside the in vivo tumor.

TIMP‐1 is secreted by various cell types in the TME. It is important to note that stromal cells such as CAFs are the primary source of TIMP‐1 in solid tumors.^58^ We found that 3D structure formation and co‐culturing with breast cancer cells could promote TIMP‐1 protein secretion in hASCs; however, the underlying mechanism by which stromal cells produce TIMP‐1 has not yet been understood. One possibility is that TIMP‐1 may be secreted when these cells are exposed to hypoxic conditions. It was previously reported that when cells encounter a hypoxic environment, hypoxia‐inducible factor 1 alpha (HIF‐1α) is activated, which upregulates the expression of TIMP‐1.^59^, ^60^ In 3D multicellular tumor model, it is well known that a gradient of oxygen levels can be established during spheroid formation and can mimic the hypoxic properties of human solid tumors. Nevertheless, the effect of HIF‐1α on TIMP‐1 expression seems to be marginal in our case, as HIF‐1α silencing in 3D tumor spheroids did not alter TIMP‐1 at either the RNA or protein level (Figure S3d–i). Whether TIMP‐1 expression is regulated by hypoxia in hASC‐co‐cultured tumor spheroids, and whether hypoxia‐related factors other than HIF‐1α might affect it remains to be elucidated.

Our results also revealed that the drug responses from cancer cells in a monolayer state may not sufficiently capture those from *in vivo* tumors. Anticancer drugs treated with IC_50_ concentrations of monolayer MDA‐MB‐231 cells showed widely different drug efficacies in 3D spheroids (Table S1). This is in line with previous studies that indicate that 3D models are better mimics of in vivo situations; Imamura et al. have reported that breast cancer spheroids in 3D culture showed higher resistance to doxorubicin compared to monolayer cells by forming dense features and inducing an antiapoptotic environment.^61^ It has been previously reported that cell packing density could affect drug penetration and resistance. Tightly packed tumor cells showed impaired penetration and relative resistance to anticancer agents compared to loosely packed cells.^62^ Fibroblast tumor spheroids seemed to be more densely packed (Figure 1a,b); however, higher drug deposition and efficacy were observed in fibroblast tumor spheroids compared to hASC‐ and hBMSC‐co‐cultured tumor spheroids (Figure 3a–d). This may indicate that the effect of cell packing on drug penetration seems to be marginal in our tumor model, and factors other than cell packing density may have had a more significant impact on it. The surrounding ECM in our 3D tumor model might play an important role in drug penetration, as in solid tumors, thereby ensuring efficient drug efficacy testing.

Despite the advantages, our model still has some limitations in simulating in vivo drug responses. Firstly, the results obtained in the tumor model using MDA‐MB‐231 breast cancer cells cannot be directly applied to other types of cancer cells. Factors such as growing conditions and drug sensitivity must be established based on tumor types. Optimally, evaluation of drug responses in a 3D tumor model generated by patient‐derived tumor and stromal cells would yield more accurate results. Secondly, the agents for assessing drug penetration and resistance were limited to small‐molecule drugs. Biologic drugs, such as antibodies and recombinant proteins, are increasingly being used in recent anticancer treatments.^63^ Thus, further studies are needed to investigate the permeability and efficacy of these biological drugs in our model. To realize this, it might be necessary to optimize 3D tumor models, including cells other than MSCs. In particular, tumor‐resident immune cells, such as macrophages, are involved in tumor progression, drug resistance, and metastasis.^64^ Therefore, we are currently developing tumor spheroids, consisting of immune cells, to more accurately analyze the efficacy of drugs related to immune responses.

Our calculations supported the notion that log*P*, PSA, and polarizability are the most effective parameters that influence intratumoral permeability, as they show both high t‐values and regression coefficients in MLR analysis. These results are acceptable because the electrostatic interactions between the drug and ECM components are known to significantly affect penetrability.^65^ The ECM mostly consists of negatively charged barriers, including collagen fibers, fibronectin, or proteoglycans, and binding or repulsion of polar drugs to these components hinders their movement through ECM.^3^ These polarity‐related input derivatives are also widely used physicochemical parameters for QSAR in drug‐likeness rules. Log*P* acts as a parameter to determine rules such as Ro5, Ghose filter, and QED, while PSA works for Veber's rule, Ghose filter, and QED.^66^ In addition, we found that drugs satisfying these rules have a higher degree of penetration measured in 3D tumor spheroids than drugs that do not meet these criteria. From this point of view, our 3D tumor model might be a reliable in vitro platform to evaluate the intratumoral penetrability of new drug candidates for solid tumors.

## 4 MATERIALS AND METHODS

### 4.1 Cell culture

hASCs were purchased from Cefobio (Seoul, Korea) and maintained in hASC growth medium (Cefobio, Seoul, Korea) supplemented 1% l‐glutamine and penicillin/streptomycin (Cefobio, Seoul, Korea). hBMSCs and hDFs were purchased from the Catholic university (Seoul, Korea), and maintained in Dulbecco's modified Eagle medium (DMEM) (Gibco, Waltham, MA, USA, Cat#11965092) supplemented with 10% fetal bovine serum (FBS, Gibco), 1% l‐glutamine and penicillin/streptomycin. A human breast carcinoma cell line, MDA‐MB‐231, was purchased from Korean Cell Line Bank (Seoul, Korea) and cultured in RPMI 1640 medium (Gibco) supplemented with 10% FBS, 1% l‐glutamine, and penicillin/streptomycin. Cells were cultured in an incubator with 5% CO_2_ at 37°C.The media were replaced every 2 days. For monolayer culture, stromal cells and breast cancer cells were seeded in 96‐well tissue culture plate (Corning, NY) with a density of 1 × 10^4^ cells /well.

### 4.2 siRNA transfection

hASCs were transfected with *HIF‐1α* (sc‐35561, Santa Cruz Biotechnology, Dallas, TX, USA) or *TIMP‐1* siRNA (sc‐29505, Santa Cruz Biotechnology) using RNAiMAX (ThermoFisher Scientific, Waltham, MA, USA) according to the manufacturer's protocol. Briefly, cells were plated at 1.5 × 10^6^ cells in T‐175 flask culture plates. Twenty‐four hours later, 25 pmol (45 μL, 10 μM) of TIMP‐1 siRNA was diluted into 2250 μL of Opti‐MEM (Gibco), and 135 μL of RNAiMAX was diluted in 2250 μL of Opti‐MEM. Diluted siRNA and RNAiMAX were then combined and incubated at room temperature for 5 min. Subsequently, 4680 μL of the siRNA‐RNAiMAX mixtures were added to T‐175 flask culture plates. Twenty‐four hours after transfection, the cells were analyzed. Knock‐down efficiency was evaluated by RT‐qPCR against HIF‐1α or TIMP‐1, respectively.

### 4.3 Preparation of nonadsorbent poly‐HEMA plates

Poly‐HEMA (Sigma Aldrich, Saint Louis, MO) was dissolved in 95% ethanol to a final concentration of 5 mg/mL, and then used for coating 96‐well U‐bottomed plate (60 μL/well). The ethanol was evaporated at 30°C incubator for a week. Poly‐HEMA‐coated plates were sterilized in UV for 1 h before culturing cells.

### 4.4 Formation of 3D spheroid

To form 3D multicellular tumor spheroids, cells was trypsinized and suspended in the growth media. Stromal cells and breast cancer cells were plated with a density of 5 × 10^3^ cells /25 μL into 96‐well plate precoated with poly‐HEMA, respectively, resulting in 1 × 10^4^ cells exist per each well. After seeding, the plate was centrifuged for 2 min at 1000 rpm to collect the cells in the center of the well. Fifty microliters of 10% Matrigel® Basement Membrane Matrix (Corning, NY, USA) solution diluted in media was gently added into the plate on ice to prevent matrigel from gelation. Monocellular spheroids were generated by a single culture of stromal cells or breast cancer cells. Cells were seeded with a density of 1 × 10^4^ cells per well with 50 μL of 10% matrigel on a poly‐HEMA‐coated plate. Then, the plate with cells and matrigel was centrifuged at 1000 rpm for 2 min, and incubated for 48 h at 37°C with 5% CO_2_.

### 4.5 Morphometric analysis

The morphology of spheroids was observed using a phase contrast microscope (Zeiss, Oberkochen, Germany). Images were analyzed by ImageJ software (National Institutes of Health, MD). Sphericity Index (SI) was computed from applying the squared root into the circularity, which is obtained from ImageJ. It quantitatively indicates how the shape of the sample is similar to the spherical geometry shape as in Equation (2).^67^

(2) $$\mathrm{SI}=\sqrt{\left(\frac{4\pi \times \text{Area}}{{\text{perimeter}}^{2}}\right).}$$

### 4.6 Scanning electron microscopy

After 2 days of 3D cell culture, 3D multicellular tumor spheroids were rinsed with phosphate‐buffered saline (PBS) three times and treated with 2.5% glutaraldehyde for 1 h at 4°C, and postfixed with 1% osmium tetroxide in deionized water for 2 h. Fixed samples were subsequently dehydrated with a series of graded ethanol (30%, 50%, 70%, 80%, 90%, and 100%) two times, 5 min at each time. After dehydration, spheroids were treated with hexamethyldisilazane for 2 min and then vacuum dried overnight. Before performing SEM, samples were transferred on the adhesive carbon tape and treated to sputter‐coating with gold for 60 s at 10 mA. The SEM images were taken at 15 kV (Inspect F50).

### 4.7 Cell labeling with tracker dye

Cells were labeled with the cell tracker dyes (Invitrogen, Carlsbad, CA, USA) according to manufacturer's instructions. Briefly, stromal cells and breast cancer cells were stained with 10 μM of CellTracker™ Green CMFDA dye and CellTracker™ Red CMTPX dye (Invitrogen), respectively, for 30 min before seeding on the plates. The distribution of cells in 3D spheroids was analyzed using a confocal microscope (LSM 700, Zeiss, Oberkochen, Germany).

### 4.8 Immunofluorescence staining

Immunofluorescence was performed as previously described.^68^, ^69^ Briefly, spheroids were harvested and fixed with 4% paraformaldehyde in PBS and embedded in optimal cutting temperature compound (Leica, Wetzlar, Germany). Then, 6 μm‐thickness‐sectioned spheroids were washed with PBS and permeabilized with 0.25% Triton X‐100 (Sigma Aldrich) in PBS for 15 min. Samples were washed three times with PBS, and blocked for 1 h at room temperature with 3% bovine serum albumin (BSA; Sigma Aldrich). Sections were incubated with primary antibodies (Table S5) diluted with blocking buffer overnight at 4°C. The samples were washed three times with PBS and incubated for 1 h with the corresponding fluorescence‐conjugated secondary antibodies (Invitrogen). Fluorescent images were observed using a confocal microscope (LSM 700, Zeiss, Oberkochen, Germany), and 3D images of spheroids were obtained by performing a maximal intensity projection of a z‐stack images.

### 4.9 RNA isolation and RT‐qPCR

Cells were harvested and homogenized using Mini‐Beadbeater‐24 (Biospec Products, Bartlesville, OK, USA). Total RNA was extracted using RNeasy Mini kit (Qiagen, Hilden, Germany) and reverse transcribed to cDNA using Superscript VILO cDNA synthesis kit (Invitrogen) according to the manufacturer's instructions. Real‐time PCR analysis was conducted using SYBR Premix Ex Taq (TaKaRa, Kusatsu, Japan) and ABI 7500 Real‐Time PCR System (Applied Biosystems, Foster City, CA) (Table S5). Gene expressions were normalized to glyceraldehyde 3‐phosphate dehydrogenase (GAPDH) expression as an internal control.

### 4.10 ELISA

Cultured cell supernatants were prepared and analyzed using quantikine human TIMP‐1 ELISA kit, human TGF‐β1 Duoset ELISA kit, human total MMP‐1 ELISA kit, and human MMP‐9 ELISA kit (R&D Systems, Minneapolis, MN, USA). ELISAs were conducted according to the manufacturer's instructions. All samples were analyzed in triplicates in each experiments.

### 4.11 MMP activity measurement

Cell‐conditioned medium was analyzed using human active MMP‐1 fluorokine E kit and human active MMP‐9 fluorokine E kit (R&D Systems, MN). The activity of MMPs was measured according to the manufacturer's protocol. All samples were analyzed in triplicates in each experiments.

### 4.12 Western blot

mmunoblotting was performed as previously described.^70^ Briefly, cultured cells were prepared and homogenized in RIPA lysis buffer (Sigma Aldrich) containing a protease and phosphatase inhibitor cocktail (Abcam, Cambridge, UK), using Mini‐Beadbeater‐24 (Biospec Products). Equal amounts of protein were then separated by 4%–15% gradient gel (Bio‐Rad Laboratories, Berkeley, CA, USA) and electrophoretically transferred to polyvinylidene fluoride membranes (Millipore, Burlington, MA, USA). The membranes were blocked with 5% BSA (Gibco) in TBST (1 M Tris–HCl, pH 7.4, 0.9% NaCl and 0.05% Tween‐20) for 1 h and probed with antibodies diluted in 3% BSA blocking solution overnight at 4°C. Membranes were then incubated with horseradish peroxidase (HRP)‐conjugated anti‐mouse or anti‐rabbit IgG (1: 100,000; Sigma Aldrich) for 1 h, and the protein bands were visualized with the enhanced chemiluminescence system (ThermoFisher Scientific), and the images were taken using iBright CL1500 imaging system (ThermoFisher Scientific).

### 4.13 Evaluation of anticancer drug efficacy and necrosis

Multicellular 3D tumor spheroids were treated with 10 μM concentrations of doxorubicin, epirubicin, and topotecan diluted with 1:1 (RPMI‐1640: DMEM) growth media for 2 days. Spheroids were transferred to an opaque‐walled multiwall plate and analyzed by ATP‐based cell viability assays using Real Time‐Glo™ MT Cell Viability Assay (Promega, Madison, WI, USA) according to the manufacturer's protocol. Drug efficacy was defined by the following Equation (3):

(3) $$\text{Drug efficacy}\%=\left(1-\text{cell viability}\right)\%=\left(1-\frac{\text{cell number of drug treated spheroid}\;}{\text{cell number of control spheroid}\;}\right)\%.$$

Necrotic cells of spheroids were analyzed in Real Time‐Glo™ Annexin V Necrosis Assay (Promega) following the manufacturer's instructions. Luminescence was measured using a Glomax discovery multi Microplate Reader (Glomax discovery, Promega) with an integration time of 0.25–1.0 s per well.

### 4.14 Calculation of PVs

Multicellular spheroids with hASC and MDA‐MB‐231, hASC, and MDA‐MB‐231 monocellular spheroids were treated with 16 anticancer drugs (Table S1), respectively, for 2 days. List of drugs used for QSAR analysis were kindly gifted from Korea Chemical Bank (Daejeon, Korea) and indicated in Table S1. Concentration of each drug treated to spheroids was equal to the IC_50_ value of MDA‐MB‐231 monolayer cells. After treating the drug with spheroids for 2 days, the efficacy of the drug was evaluated by CellTiter‐Glo® 3D Cell Viability Assay (Promega). PVs were calculated as follows, where Eff_A:M_ is a drug efficacy of Multicellular spheroids with hASC and MDA‐MB‐231, Eff_ASC_ is a drug efficacy of hASC monocellular spheroids, and Eff_MDA_ is a drug efficacy of MDA‐MB‐231 monocellular spheroids.

(4) $$\mathrm{PV}={\mathrm{Eff}}_{A:M}-\frac{1}{2}\;\left({\mathrm{Eff}}_{\mathrm{ASC}}+{\mathrm{Eff}}_{\mathrm{MDA}}\right).$$

### 4.15 Calculation of Caco‐2 permeability and PAMPA

Caco‐2 cell PV for each drug was obtained from the Caco‐2 permeable value of Predicted ADMET Features listed in the DrugBank (https://go.drugbank.com/) and admetSAR (http://lmmd.ecust.edu.cn/admetsar2). The drug's PAMPA PV was calculated through the following equation as previously reported,^71^ where log*P* is a partition coefficient, p*K*_a_ is an acid dissociation constant, SA_HA_ is surface area occupied by hydrogen bond acceptor atoms, and SA_HD_ is surface area occupied by hydrogen bond donor atoms.

(5) $$\log P_{\text{PAMPA}}=0.42\times \log P-0.26\times |pK_{a}-\mathrm{pH}|-1.11\times \left(SA_{\mathrm{HA}}\right)-1.01\times \left(SA_{\mathrm{HD}}\right)-4.93.$$

### 4.16 Statistical analysis

All values are represented as mean ± *SEM* from two or more independent experiments. Statistical significance was determined using unpaired student's *t*‐test or one‐way analysis of variance (ANOVA) followed by Bonferroni's multiple comparison tests, provided by the GraphPad Prism 7 (GraphPad Software, San Diego, CA, USA) software. Statistical significance was defined as *p < 0.05, **p < 0.01, and ***p < 0.001, respectively. Multiple regression analysis was performed by stepwise regression using JMPpro statistical analysis software (SAS Institute, Cary, NC, USA). For the selection of the most relevant descriptors, forward stepwise variable selection method was applied.

## 5 CONCLUSION

In summary, we successfully developed 3D multicellular tumor spheroids by co‐culturing hASCs and breast cancer cells. This model could effectively mimic the surrounding ECM and drug‐resistant characteristics of in vivo tumors by overexpressing TIMP‐1. For reproducible drug screening, monolayer culture systems may be inaccurate because they cannot reflect the physiologic configuration of cells that exists in a 3D environment in vivo. Other approaches, such as cancer‐omics analysis or patient‐derived xenograft, have several unsolved limitations that require time‐consuming procedures and vast resources.^2^ In this situation, 3D in vitro models could be suitable alternatives to meet drug screening efficiency, efficacy, and developmental costs. Further studies are warranted to investigate the possibility of using this 3D multicellular tumor model, particularly spheroids made of cancer and stromal cell clusters, to screen therapeutics that could efficiently treat solid tumors.

## CONFLICT OF INTEREST

The authors declare no conflict of interest.

## AUTHOR CONTRIBUTIONS

**Sang‐Heon Kim:** Conceptualization (equal); funding acquisition (lead); methodology (equal); project administration (lead); supervision (lead); writing – original draft (equal); writing – review and editing (equal). **In Yeong Bae:** Conceptualization (equal); formal analysis (equal); investigation (equal); methodology (equal); validation (equal); visualization (equal); writing – original draft (equal). **Wooshik Choi:** Conceptualization (equal); formal analysis (equal); investigation (equal); visualization (equal); writing – original draft (equal); writing – review and editing (supporting). **Seung Ja Oh:** Conceptualization (equal); supervision (supporting). **Chansoo Kim:** Supervision (supporting); writing – review and editing (equal).

### PEER REVIEW

The peer review history for this article is available at https://publons.com/publon/10.1002/btm2.10286.

## Supporting information


**FIGURE S1** ECM markers and MMPs expression in 3D monocellular spheroids
**FIGURE S2**. TGF‐β1, TIMP‐1 expression, and cell distribution in the 3D multicellular tumor spheroids
**FIGURE S3**. Regulations of TIMP‐1 expression in the 3D spheroids.
**FIGURE S4**. Effects of anticancer drugs on the survival of 3D monocellular spheroids
**TABLE S1**. List of 16 drugs and its IC50 values in MDA‐MB‐231 cells and efficacy in spheroids
**TABLE S2**. List of drug likeness rules and PAMPA, Caco‐2 permeability values of 16 drugs
**TABLE S3**. List of experimental and predicted permeability values (PVs) of 16 drugs derived from MLR analysis
**TABLE S4**. List of input and output variants of sixteen drugs for MLR analysis
**TABLE S5**. Primers and antibodies used in this study

## Data Availability

Data sets generated and/or analyzed during this study are available from the corresponding author on reasonable request.
