# Supplementary material for: TIMP‐1‐expressing breast tumor spheroids for the evaluation of drug penetration and efficacy
Source: Bioeng Transl Med. 2021 Dec 31;7(2):e10286. doi: 10.1002/btm2.10286 (PMC9115709; doi:10.1002/btm2.10286)
Supplement: Supplementary file 1 — FIGURE S1 ECM markers and MMPs expression in 3D monocellular spheroids FIGURE S2. TGF‐β1, TIMP‐1 expression, and cell distribution in the 3D multicellular tumor spheroids FIGURE S3. Regulations of TIMP‐1 expression in the 3D spheroids. FIGURE S4. Effects of anticancer drugs on the survival of 3D monocellular spheroids TABLE S1. List of 16 drugs and its IC50 values in MDA‐MB‐231 cells and efficacy in spheroids TABLE S2. List of drug likeness rules and PAMPA, Caco‐2 permeability values of 16 drugs TABLE S3. List of experimental and predicted permeability values (PVs) of 16 drugs derived from MLR analysis TABLE S4. List of input and output variants of sixteen drugs for MLR analysis TABLE S5. Primers and antibodies used in this study [file BTM2-7-e10286-s001.pdf]

Supplementary material

Fig. S1

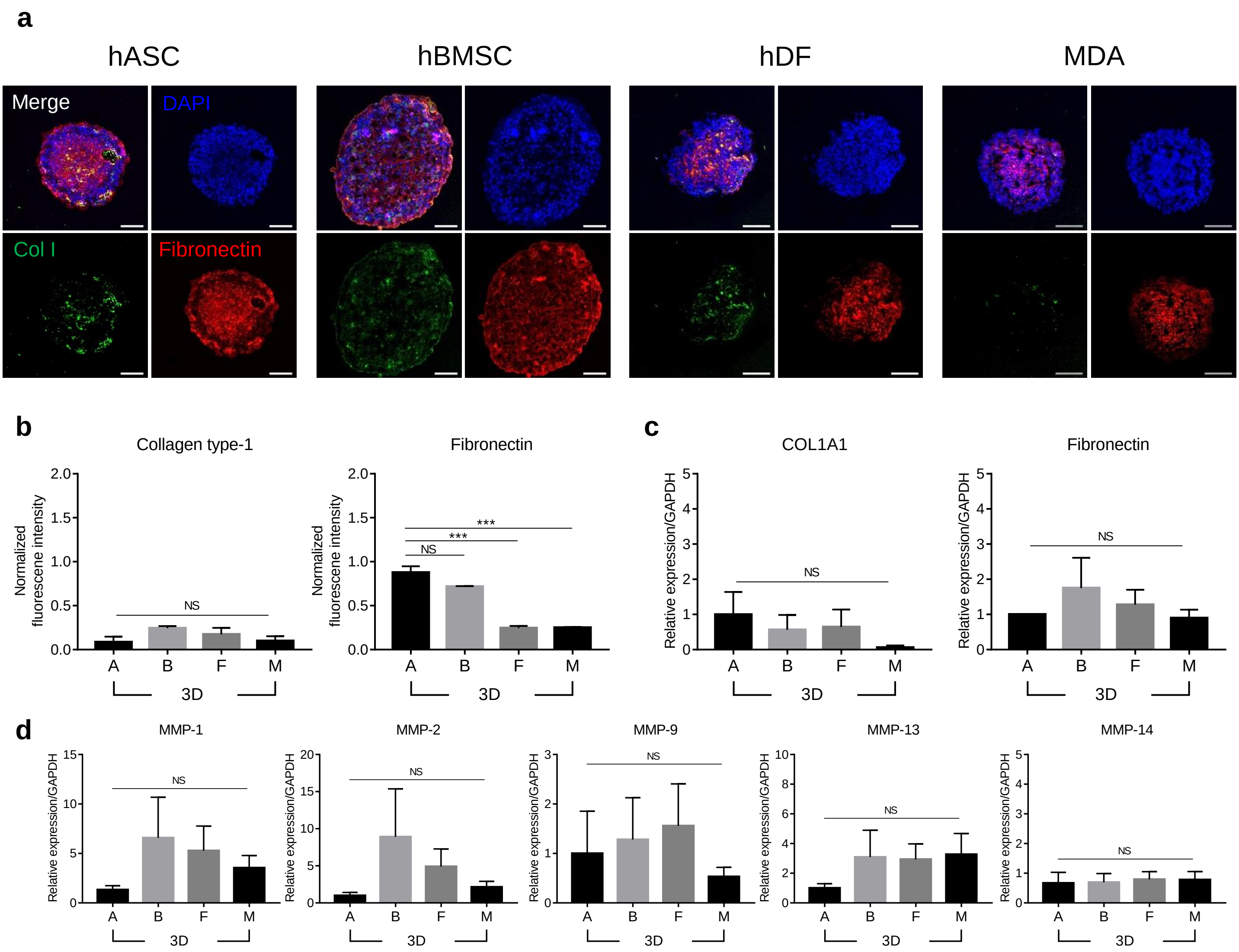

Fig. S1. ECM markers and MMPs expression in 3D monocellular spheroids

Stromal cells and the breast cancer cells were cultured respectively to form 3D monocellular spheroids for 48 h. (A) Collagen type-1 (green) and fibronectin (red) staining of monocellular spheroids. Scale bars, 100 µm. (B) Quantifications of fluorescence intensity for collagen type-1 and fibronectin in the monocellular spheroids. Values were normalized to the intensity of DAPI. NS, not significant, ***p<0.001 (one-way ANOVA), n=3 per group. mRNA expression of (C) collagen type-1 alpha 1, fibronectin, and (D) MMPs in the monocellular spheroids. NS, not significant, n=3 per group. For RT-qPCR analysis, values were normalized to GAPDH. All data are presented as mean ± SEM.

Fig. S2

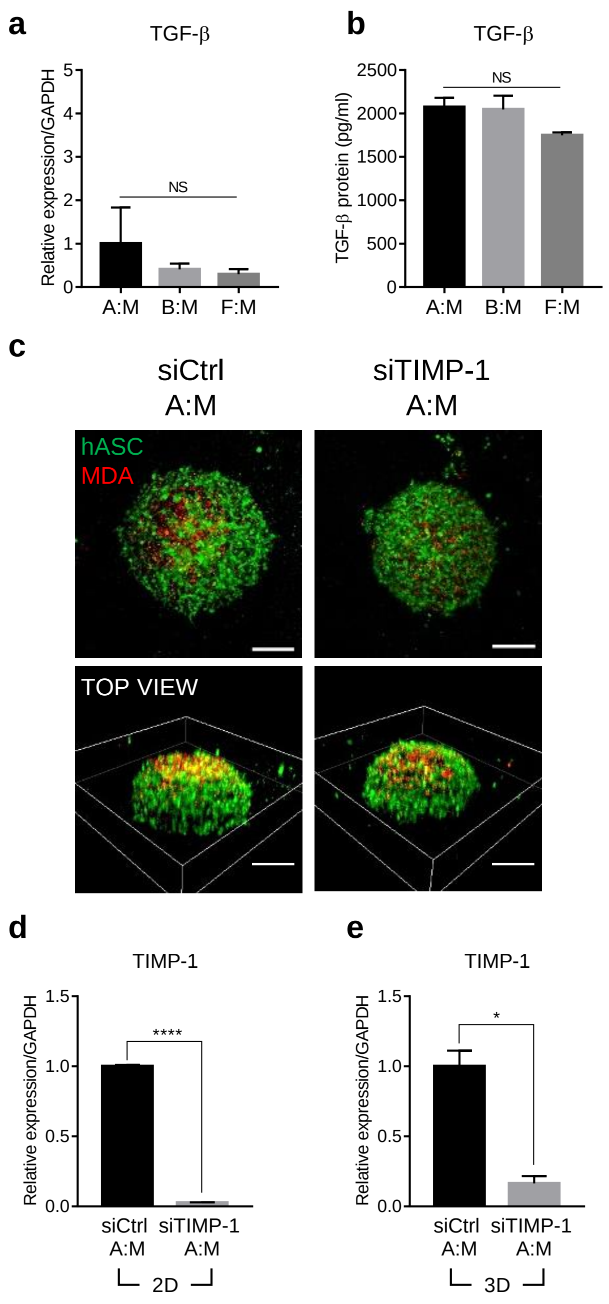

Fig. S2. TGF-β1, TIMP-1 expression and cell distribution in the 3D multicellular tumor spheroids

RNA or conditioned media from the tumor spheroids were prepared followed by RT-qPCR or ELISA, respectively. (A) mRNA expression and (B) Protein secretion of TGF-β in the tumor spheroids. NS, not significant (one-way ANOVA), n=3 per group. (C-E) hASCs were transfected with TIMP-1 or control siRNAs, and the RNA from the monolayer cells or tumor spheroids were isolated and subjected to RT-qPCR. (C) Distribution of breast cancer cells (red) and hASCs (green) in the tumor spheroids. Scale bars, 200 μm. Knockdown efficiency of TIMP-1 in (D) monolayer cultures and in (E) 3D tumor spheroids. *$p<0.05$, ****$p<0.0001$ (unpaired student's t test), n=3 per group. All data are presented as mean ± SEM.

Fig. S3

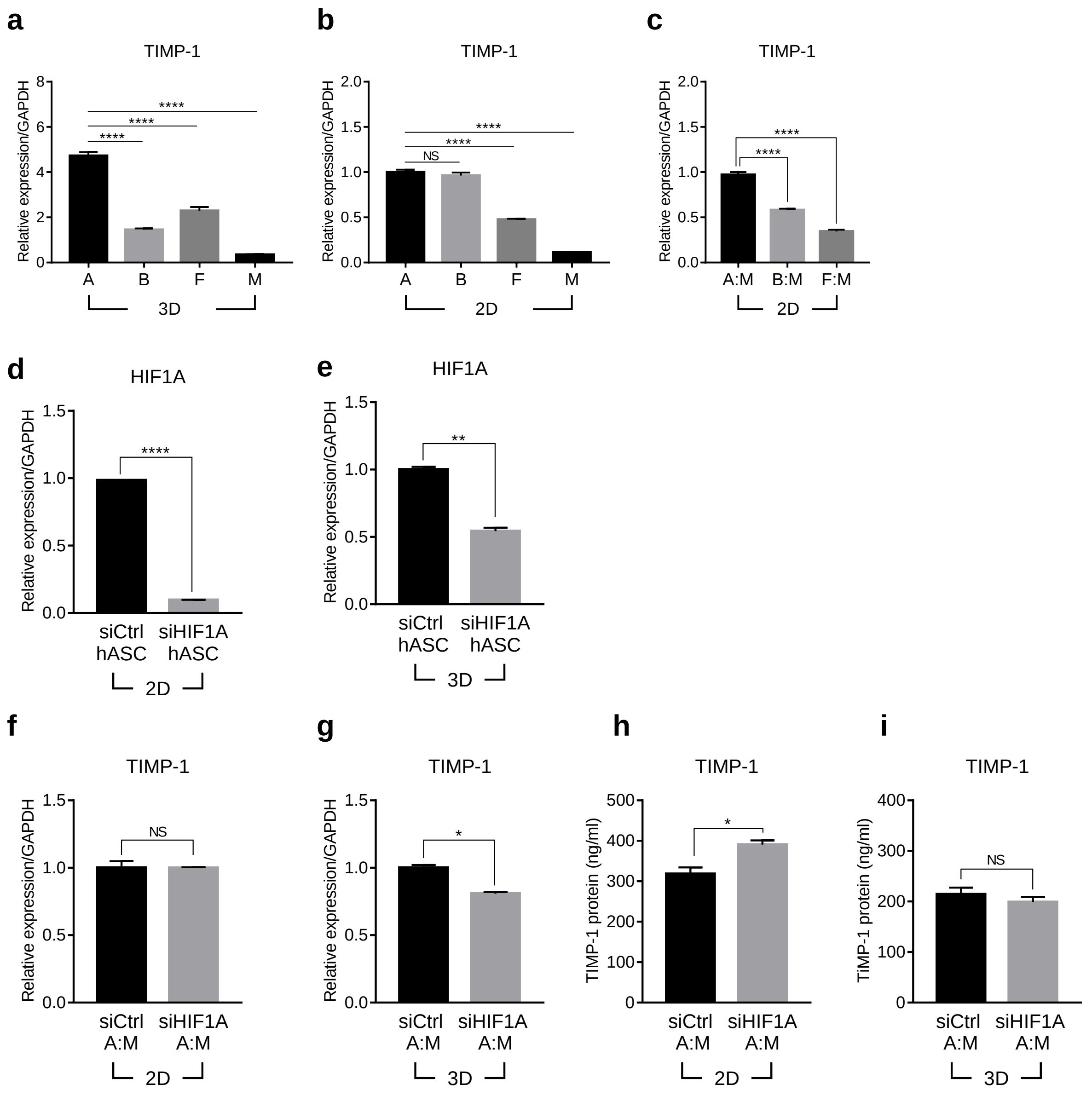

Fig. S3. Regulations of TIMP-1 expression in the 3D spheroids.

TIMP-1 mRNA expression in (A) 2D or (B) 3D cultured stromal cells and breast cancer cells. NS, not significant, ****$p<0.0001$ (one-way ANOVA), n=3 per group. (C) TIMP-1 mRNA expression in 2D co-cultured stromal cells and breast cancer cells. ****$p<0.0001$ (one-way ANOVA), n=3 per group. (D-I) hASCs were transfected with HIF-1α or control siRNAs, and 2D or 3D co-cultured with breast cancer cells for 48 h. Knockdown efficiency of HIF-1α in (D) monolayer hASCs and in (E) hASC monocellular spheroids. **$p<0.01$, ****$p<0.0001$ (unpaired student's t test), n=3 per group. Effect of HIF-1α on the TIMP-1 mRNA expression in (F) monolayer cultures and in (G) 3D tumor spheroids. NS, not significant, *$p<0.05$ (unpaired student's t test), n=3 per group. Effect of HIF-1α on TIMP-1 protein secretion in (H) monolayer cultures and in (I) 3D tumor spheroids. NS, not significant, *$p<0.05$ (unpaired student's t test), n=3 per group. For RT-qPCR analysis, values were normalized to GAPDH. All data are presented as mean ± SEM.

Fig. S4

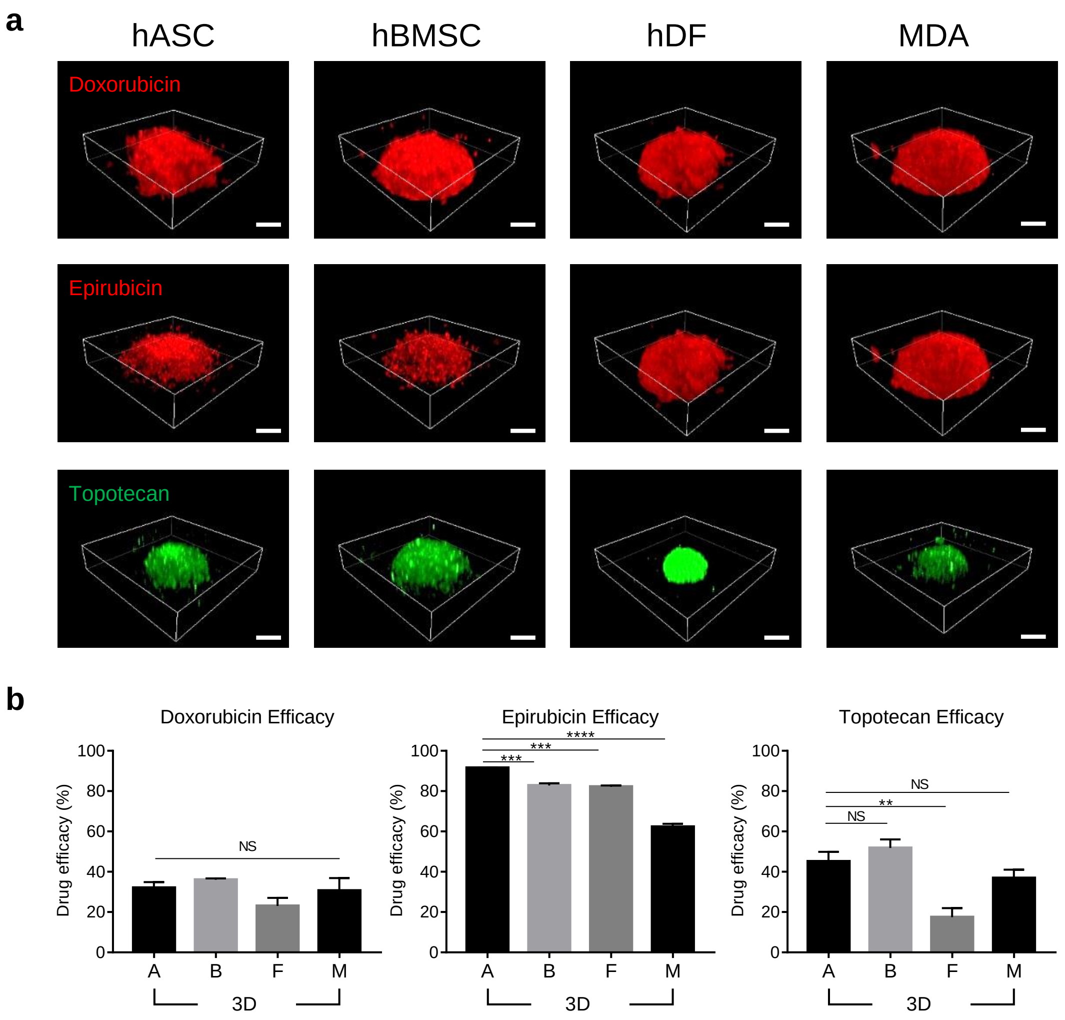

Fig. S4. Effects of anti-cancer drugs on the survival of 3D monocellular spheroids

Stromal cells and the breast cancer cells were cultured respectively to form 3D monocellular spheroids for 48 h, and anti-cancer drugs were treated to the spheroids for an additional 48 h. (A) Disposition of doxorubicin, epirubicin, and topotecan penetrated into the monocellular spheroids. Scale bars, 100 μm. (B) Efficacy of anti-cancer drugs in the monocellular spheroids. NS, not significant, **p<0.01, ***p<00001, ****p<0.0001 (one-way ANOVA), n=3 per group. All data are presented as mean ± SEM.

Table S1

	Drug	MDA-MB-231 IC_{50} (μM)	A:M spheroid Efficacy ($Eff_{A:M}$, %)	hASC spheroid Efficacy (Eff_{ASC}, %)	MDA spheroid Efficacy (Eff_{MDA}, %)
1	Ammonium-glycyrrhizinate	72.143	27.510	33.753	40.204
2	Etoposide	32.580	72.013	73.263	64.897
3	Capecitabine	95.020	99.932	99.895	99.873
4	Paclitaxel	2.380	80.377	97.404	72.690
5	Rubitecan	0.820	19.215	17.266	25.373
6	Mitomycin-c	17.480	51.771	64.309	53.401
7	Bardoxolone-methyl	2.160	-8.940	38.156	-35.997
8	Epirubicin	1.569	28.973	45.548	23.696
9	Talazoparib	87.420	92.742	99.742	75.508
10	Patupilone (epothilone-b)	33.420	25.320	34.560	23.781
11	SN38 (7-ethyl-10-hydroxycamptothecin)	1.870	62.563	60.449	58.491
12	Vinblastine	1.830	11.251	25.299	9.274
13	Rucaparib	72.344	96.300	99.818	92.185
14	Altretamine	64.450	41.432	41.038	36.883
15	Verubulin	0.010	20.146	24.939	5.173
16	Mifepristone	15.406	60.879	91.524	46.672

Table S1. List of sixteen drugs and its IC50 values in MDA-MB-231 cells and efficacy in spheroids.

Table S2

	Drug	Rule of five (Ro5)	Ghose filter	Veber's rule	Unweighted QED	*log*P_{PAMPA}	Caco-2 cell permeability	Experiment’s PV #1	Experiment’s PV #2	Experiment’s PV #3
1	ammonium-glycyrrhizinate	No	No	No	No	-9.511	-	-0.114	-0.075	-0.095
2	etoposide	No	No	No	No	-9.236	-	0.029	0.043	0.016
3	capecitabine	Yes	Yes	No	No	-8.572	-	0.000	0.001	0.000
4	paclitaxel	No	No	No	No	-8.137	-	-0.047	-0.071	-0.023
5	rubitecan	Yes	Yes	No	No	-7.309	-	-0.006	-0.036	-0.021
6	mitomycin-c	Yes	No	No	No	-7.148	-	-0.071	-0.097	-0.045
7	bardoxolone-methyl	No	No	No	No	-6.919	-	-0.100	-0.171	-0.030
8	epirubicin	No	No	No	No	-6.556	-	-0.096	-0.017	-0.056
9	talazoparib	Yes	Yes	No	No	-6.076	+	0.051	0.057	0.046
10	Patupilone(epothilone-b)	No	No	No	No	-5.750	-	-0.039	-0.067	-0.010
11	SN38 (7-ethyl-10-hydroxycamptothecin)	Yes	Yes	No	Yes	-5.746	-	0.031	0.035	0.027
12	vinblastine	No	No	No	Yes	-4.830	+	-0.060	-0.020	-0.100
13	rucaparib	Yes	Yes	No	Yes	-4.524	-	0.004	0.002	0.003
14	altretamine	Yes	Yes	No	Yes	-4.447	+	0.025	0.062	-0.012
15	Verubulin	Yes	Yes	Yes	Yes	-4.372	+	0.039	0.029	0.085
16	mifepristone	No	No	No	Yes	-3.758	+	-0.082	-0.048	-0.116

Table S2. List of drug likeness rules and PAMPA, Caco-2 permeability values of sixteen drugs

Table S3

	Drug	INPUT									OUTPUT			Predicted PV
											Experiment's PV			
		MW	LOGP	LOGS	Hydrogen Acceptor Count	Hydrogen Donor Count	Polar Surface Area (Å2)	Rotatable Bond Count	Refractivity (m3·mol-1)	Polarizability (Å3)	#1	#2	#3	predicted permeability
1	Ammonium-glycyrrhizinate	840	2.84	-4.3	16	7	269.87	7	209.67	85.75	-0.114	-0.075	-0.095	-0.098715177
2	Etoposide	588.6	0.73	-2.8	12	3	160.83	5	139.02	58.77	0.029	0.043	0.016	0.039695671
3	Capecitabine	359.35	1.17	-3.2	6	3	120.69	7	82.75	35.81	0.000	0.001	0.000	0.024631629
4	Paclitaxel	853.906	3.2	-5.2	10	4	221.29	14	218.29	87.17	-0.047	-0.071	-0.023	-0.059652167
5	Rubitecan	393.3	1.88	-3.2	6	1	122.87	2	100.81	38.87	-0.006	-0.036	-0.021	-0.010709304
6	Mitomycin-c	334.332	-0.55	-1.5	7	3	146.89	4	83.27	32.77	-0.071	-0.097	-0.045	-0.064332469
7	Bardoxolone-methyl	505.7	5.29	-5.2	4	0	84.23	2	144.36	58.23	-0.100	-0.171	-0.030	-0.081853259
8	Epirubicin	543.525	1.41	-2.7	12	6	206.07	5	134.59	53.88	-0.096	-0.017	-0.056	-0.046591856
9	Talazoparib	380.35	2.93	-3.6	5	2	84.2	2	111.27	35.82	0.051	0.057	0.046	0.059519503
10	Patupilone (epothilone-b)	507.7	3.7	-5.2	6	2	109.25	2	134.76	56.11	-0.039	-0.067	-0.010	-0.068215634
11	SN38(7-ethyl-10-hydroxycamptothecin)	392.404	2.73	-3.1	5	2	99.96	2	106.12	41.43	0.031	0.035	0.027	0.001029053
12	Vinblastine	810.975	4.22	-4.7	9	3	154.1	10	222.42	87.46	-0.060	-0.020	-0.100	-0.061434873
13	Rucaparib	323.4	2.39	-4.4	2	3	56.92	3	92.91	35.19	0.004	0.002	0.003	-0.01221618
14	Altretamine	210.28	2.43	-1.8	6	0	48.39	3	65.65	23.7	0.025	0.062	-0.012	0.009735848
15	Verubulin	279.34	3.8	-3.6	4	0	38.25	3	83.58	31.04	0.039	0.029	0.085	0.032210289
16	Mifepristone	429.6	5.33	-5.1	3	1	40.54	3	132.58	50.69	-0.082	-0.048	-0.116	-0.043616451

Table S3. List of experimental and predicted permeability values (PVs) of sixteen drugs derived from MLR analysis.

Table S4

	Drug	INPUT													OUTPUT		
															Experiment's PV		
		M.W	water Solubility (mg/mL)	logP	logS	pKa (Strongest Basic)	Physiological Charge	Hydrogen Acceptor Count	Hydrogen Donor Count	Polar Surface Area (Å2)	Rotatable Bond Count	Refractivity (m3·mol-1)	Polarizability (Å3)	Number of Rings	#1	#2	#3
1	Ammonium-glycyrrhizinate	840	0.0427	2.84	-4.3	-3.7	-3	16	7	269.87	7	209.67	85.75	7	-0.114	-0.075	-0.095
2	Etoposide	588.6	0.978	0.73	-2.8	-3.7	0	12	3	160.83	5	139.02	58.77	7	0.029	0.043	0.016
3	Capecitabine	359.35	0.248	1.17	-3.2	-3.6	0	6	3	120.69	7	82.75	35.81	2	0.000	0.001	0.000
4	Paclitaxel	853.906	0.00556	3.2	-5.2	-1	0	10	4	221.29	14	218.29	87.17	7	-0.047	-0.071	-0.023
5	Rubitecan	393.3	0.239	1.88	-3.2	0.29	0	6	1	122.87	2	100.81	38.87	5	-0.006	-0.036	-0.021
6	Mitomycin-c	334.332	10.1	-0.55	-1.5	5.76	0	7	3	146.89	4	83.27	32.77	4	-0.071	-0.097	-0.045
7	Bardoxolone-methyl	505.7	0.00294	5.29	-5.2	-5.3	0	4	0	84.23	2	144.36	58.23	5	-0.100	-0.171	-0.030
8	Epirubicin	543.525	1.18	1.41	-2.7	8.94	1	12	6	206.07	5	134.59	53.88	5	-0.096	-0.017	-0.056
9	Talazoparib	380.35	0.101	2.93	-3.6	1.66	0	5	2	84.2	2	111.27	35.82	5	0.051	0.057	0.046
10	Patupilone (epothilone-b)	507.7	0.00342	3.7	-5.2	2.73	0	6	2	109.25	2	134.76	56.11	3	-0.039	-0.067	-0.010
11	SN38 (7-ethyl-10-hydroxycamptothecin)	392.404	0.29	2.73	-3.1	3.91	0	5	2	99.96	2	106.12	41.43	5	0.031	0.035	0.027
12	Vinblastine	810.975	0.0169	4.22	-4.7	8.86	2	9	3	154.1	10	222.42	87.46	9	-0.060	-0.020	-0.100
13	Rucaparib	323.4	0.0114	2.39	-4.4	9.32	1	2	3	56.92	3	92.91	35.19	4	0.004	0.002	0.003
14	Altretamine	210.28	3.1	2.43	-1.8	7.75	1	6	0	48.39	3	65.65	23.7	1	0.025	0.062	-0.012
15	Verubulin	279.34	0.0641	3.8	-3.6	4.94	0	4	0	38.25	3	83.58	31.04	3	0.039	0.029	0.085
16	Mifepristone	429.6	0.00336	5.33	-5.1	4.89	0	3	1	40.54	3	132.58	50.69	5	-0.082	-0.048	-0.116

Table S4. List of input and output variants of sixteen drugs for MLR analysis.

Table S5
(A)

Primers	Forward Sequence (5’-3’)	Reverse Sequence (5’-3’)
MMP1	GATGGACCTGGAGGAAATCTTG	TGAGCATCCCCTCCAATACC
MMP2	GCACCCATTTACACCTACACCAA	AGAGCTCCTGAATGCCCTTGA
MMP9	GGACGATGCCTGCAACGT	ACAAATACAGCTGGTTCCCAATC
MMP13	TTCTTGTTGCTGCGCATGA	TGCTCCAGGGTCCTTGGA
MMP14	CCGATGTGGTGTTCCAGACA	TCGTATGTGGCATACTCGCC
TIMP1	GTTGTTGCTGTGGCTGATAG	TGTGGGACCTGTGGAAGTA
Col1a1	CAGCCGCTTCACCTACAGC	TTTTGTATTCAATCACTGTCTTGCC
Fibronectin	TGGACCAGAGATCTTGGATGTTC	CGCCTAAAACCATGTTCCTCAA
HIF1a	CCAGTTACGTTCCTTCGATCAGTTG	AGCAGTAGGTTCTTGTATTTGAGTCTG
GAPDH	AAGGCTGTGGGCAAGG	TGGAGGAGTGGGTGTCG

(B)

Antibody	Host	Company	Catalog	Notes
Collagen I	Mouse	Abcam	ab90395	WB (1:500), IF (1:200)
Fibronectin	Rabbit	Abcam	ab23750	WB (1:500), IF (1:200)
GAPDH	Mouse	Abcam	ab8245	WB (1:5000)
IgG-Alexa Fluor488	Mouse	ThermoFisher Scientific	A11001	IF (1:500)
IgG-Alexa Fluor594	Rabbit	ThermoFisher Scientific	A11012	IF (1:500)

Table S5. Primers and antibodies used in this study

(A) Nucleotide sequences of primer used for RT-qPCR analysis. (B) Antibodies used for Western blot hybridization and immunofluorescence.
